# LYVE-1–expressing Macrophages Modulate the Hyaluronan-containing Extracellular Matrix in the Mammary Stroma and Contribute to Mammary Tumor Growth

**DOI:** 10.1158/2767-9764.CRC-24-0205

**Published:** 2024-05-31

**Authors:** Alexis K. Elfstrum, Annisa H. Rumahorbo, Lyndsay E. Reese, Emma V. Nelson, Braedan M. McCluskey, Kathryn L. Schwertfeger

**Affiliations:** 1 Microbiology, Immunology, and Cancer Biology Graduate Program, University of Minnesota, Minneapolis, Minnesota.; 2 Department of Laboratory Medicine and Pathology, University of Minnesota, Minneapolis, Minnesota.; 3 University of Minnesota Supercomputing Institute, University of Minnesota, Minneapolis, Minnesota.; 4 Masonic Cancer Center, University of Minnesota, Minneapolis, Minnesota.; 5 Center for Immunology, University of Minnesota, Minneapolis, Minnesota.

## Abstract

Macrophages represent a heterogeneous myeloid population with diverse functions in normal tissues and tumors. While macrophages expressing the cell surface marker lymphatic vessel endothelial hyaluronan receptor 1 (LYVE-1) have been identified in stromal regions of the normal mammary gland and in the peritumoral stroma, their functions within these regions are not well understood. Using a genetic mouse model of LYVE-1^+^ macrophage depletion, we demonstrate that loss of LYVE-1^+^ macrophages is associated with altered extracellular matrix remodeling in the normal mammary gland and reduced mammary tumor growth *in vivo*. In further studies focused on investigating the functions of LYVE-1^+^ macrophages in the tumor microenvironment, we demonstrate that LYVE-1 expression correlates with an increased ability of macrophages to bind, internalize, and degrade hyaluronan. Consistent with this, we show that depletion of LYVE-1^+^ macrophages correlates with increased hyaluronan accumulation in both the normal mammary gland and in mammary tumors. Analysis of single-cell RNA sequencing of macrophages isolated from these tumors reveals that depletion of LYVE-1^+^ macrophages in tumors drives a shift in the majority of the remaining macrophages toward a proinflammatory phenotype, as well as an increase in CD8^+^ T-cell infiltration. Together, these findings indicate that LYVE-1^+^ macrophages represent a tumor-promoting anti-inflammatory subset of macrophages that contributes to hyaluronan remodeling in the tumor microenvironment.

**Significance:**

We have identified a macrophage subset in mouse mammary tumors associated with tumor structural components. When this macrophage subset is absent in tumors, we report a delay in tumor growth and an increase in antitumor immune cells. Understanding the functions of distinct macrophage subsets may allow for improved therapeutic strategies for patients with breast cancer.

## Introduction

Macrophages are a heterogeneous population of cells, encompassing an array of phenotypically distinct subsets (1). The functions of macrophage subsets vary in support or inhibition of tumor growth. For example, macrophages produce immunosuppressive cytokines such as IL10 and TGFβ, which support regulatory T cells and subsequently inhibit tumor-infiltrating lymphocytes (2, 3). In another tumor-supporting role, macrophages produce matrix metalloproteases (MMP), which contribute to tumor progression by remodeling the extracellular matrix (ECM; refs. 4, 5). In addition, macrophages can directly internalize and degrade collagen in the dermis via cluster of differentiation 206 (CD206; ref. 6). Conversely, macrophages are also capable of releasing cytotoxic molecules such as reactive oxygen species (ROS), which are toxic to tumor cells and can also recruit tumor inhibitory immune cells such as CD8 T cells and natural killer (NK) cells^,^ (7). Recent studies have demonstrated substantial phenotypic heterogeneity of tumor-associated macrophages (8, 9), suggesting that multiple phenotypic subsets of macrophages exist within the tumor microenvironment. Identifying and defining the functions of these macrophage subsets are important for understanding their contributions to tumor progression.

Recent studies have identified lymphatic vessel endothelial hyaluronan receptor-1 (LYVE-1), a transmembrane scavenger receptor lacking enzymatic activity (10), as a marker of a distinct subset of interstitial macrophages (11). LYVE-1 was first identified as a marker of lymphatic endothelial cells (LEC) and was later linked to a subset of resident macrophages that have been found in numerous tissues including the lung and aorta (11–13). Within these tissues, LYVE-1^+^ macrophages have been shown to regulate tissue homeostasis via various mechanisms, including suppression of fibrosis in the lung and maintenance of arterial vessels in the heart (11, 13). LYVE-1^+^ macrophages have also been identified in breast and other cancer types. For example, macrophages expressing LYVE-1 and other LEC-related markers, such as podoplanin (PDPN), have been shown to localize to lymphatic regions and contribute to lymphangiogenesis in various tumor models (14). Furthermore, perivascular LYVE-1^+^ macrophages have also been identified in the mouse mammary tumor virus (MMTV)-polyoma middle T (PyMT) spontaneous mammary tumor model and been shown to contribute to regulation of the angiogenic niche (15). In addition, LYVE-1^+^ macrophages of the peritoneal mesothelium have been found to promote growth in ovarian tumors (16). We and others have identified LYVE-1^+^ macrophages at the tumor periphery in mouse models of melanoma and breast cancer, although the functions of these peritumoral macrophages have not been investigated (17, 18).

LYVE-1 is a receptor for the glycosaminoglycan hyaluronic acid (HA; ref. 19). HA serves as a structural component of the ECM in normal tissues and in tumors and can be synthesized by various cell types including tumor cells and fibroblasts (20). In LECs, LYVE-1 binds and endocytoses HA, which can then be degraded in the lysosome by hyaluronidases (21–23). While the role of LYVE-1–expressing macrophages in regulating HA has not been previously investigated in the context of tumor-associated macrophages, we have shown that LYVE-1^+^ macrophages localize to HA-enriched regions in the normal mammary gland. Furthermore, we demonstrated these macrophages exhibit gene profiles that are associated with glycosaminoglycan binding and tissue remodeling (18). A key aspect of tissue remodeling is the breakdown of ECM components such as HA or collagen. Mechanistically, tumor-associated macrophages are known to secrete extracellular proteases such as MMPs, which degrade ECM components (24). ECM degradation clears space within the intratmoral and peritumoral regions, allowing for tumor cells to migrate to blood vessels and lymphatics and to release growth factors that can be used by tumor cells during tumor progression (4, 25, 26). While there is an established link between tumor-associated macrophages and protease-mediated ECM degradation, less is known regarding the ability of tumor-associated macrophages to modulate key non-proteinaceous components of the ECM, such as HA.

Here, we examine the phenotype of normal and cancerous mammary tissue using a genetic mouse model in which LYVE-1^+^ macrophages are selectively depleted. We demonstrate that LYVE-1^+^ macrophage depletion results in HA accumulation in both the nulliparous mammary gland and in mammary tumors, which correlates with a decrease in mammary tumor growth. In addition, we show that LYVE-1^+^ macrophages are associated with HA-enriched regions at the tumor periphery, outside of the tumor parenchyma. Furthermore, we demonstrate that LYVE-1^+^ macrophages are associated with ECM remodeling in both normal and malignant mammary tissue using *in vitro* assays and single-cell RNA sequencing (scRNA-seq). Further analysis of scRNA-seq data reveals that in the absence of LYVE-1^+^ macrophages, three of the four remaining macrophage clusters shift toward a proinflammatory phenotype. In addition, more CD8^+^ T cells are found in tumors lacking LYVE-1^+^ macrophages, suggesting that LYVE-1^+^ macrophages have anti-inflammatory functions within the tumor microenvironment. Taken together, our findings demonstrate that LYVE-1^+^ macrophages contribute to ECM remodeling and represent a subset of tumor supportive macrophages that may provide a novel target for breast cancer treatment.

## Materials and Methods

### Mice

Mice were purchased from Jackson Laboratories (Wild Type C57BL/6J, Stock #000664; *Lyve1^Cre^* C57BL/6, Stock #012601; *Csf1r^fl/fl^* C57BL/6, Stock #021212) and *Lyve1^Cre^* and *Csf1r^fl/fl^* mice were crossed in house. Animal care and procedures were approved by the Institutional Animal Care and Use Committee of the University of Minnesota (Minneapolis, MN) in accordance with the procedures detailed in the Guide for the Care and Use of Animals.

### Cell Culture

J774 and EO771 cell lines were cultured per ATCC recommendations and subjected to *Mycoplasma* testing (MycoAlert, VWR).

### Mammary Gland Isolation and Flow Cytometry

For analysis of immune cells within mammary glands, inguinal (#4) mammary glands were harvested from female 5-week and 12-week mice with inguinal lymph nodes removed. Mammary glands were digested with 1 mg/mL collagenase (Sigma-Aldrich), 400 U/mL hyaluronidase (Sigma-Aldrich), and 15 µg/mL DNase I (Sigma-Aldrich) for 45 minutes with shaking at 37°C. The digested mammary glands were filtered through a 70 µm cell strainer and pelleted by centrifugation at 300 × *g* for 5 minutes. Red blood cells were lysed with buffer containing 150 mmol/L ammonium chloride, 10 mmol/L potassium bicarbonate, and 0.1 mmol/L sodium Ethylenediaminetetraacetic acid (EDTA) at pH 7.4, and then resuspended in FACS buffer (PBS containing 2% FBS and 1 mmol/L EDTA). Cells were stained for extracellular markers in FACS buffer at room temperature and fixable viability dye (eBioscience) was used to identify viable cells. Fluorochrome conjugated primary antibodies were used to identify CD45 (BD Biosciences 30-F11), F4/80 (BioLegend BM8), CD11b (BD Biosciences M1/70), LYVE-1 (R&D Systems 223322), TIM4 (BioLegend F31-5G3), and Podoplanin (eBioscience 8.1.1). An antibody that is specific to mouse CD16 and CD32 (eBioscience, clone 93) was included to block Fc Receptor. Cells were fixed using 1% paraformaldehyde for 30 minutes at 4°C. Cells were permeabilized for 5 minutes at room temperature using 1X Flow Cytometry Perm Buffer (Tonbo biosciences). Samples were stained for intracellular protein CD206 (BioLegend C068C2) in 1X Perm Buffer. Flow cytometry was performed using an LSR Fortessa X-20 (BD Biosciences) and analyzed with FlowJo Software. t-distributed stochastic neighbor embedding (t-SNE) plots of the F4/80^+^CD11b^+^ population population were made in FlowJo software using CD206, PDPN, and TIM4 as parameters.

To examine HA binding *in vivo,* mammary glands from female 5-week-old wild-type C57BL/6 mice were harvested, dissociated, and prepared for flow cytometry as described above. Cells were stained for extracellular markers CD45 (BD Biosciences 30-F11), F4/80 (BioLegend BM8), CD11b (BD Biosciences M1/70), LYVE-1 (R&D Systems 223322), and biotinylated HA-binding peptide (HABP, VWR 80502-722), followed by streptavidin conjugated BV711 (BioLegend 405241) secondary antibody. HA binding was determined by mean fluorescent intensity of LYVE-1^−^ and LYVE-1^+^ macrophages.

### Immunostaining

For identification of macrophages within mammary glands, #4 mammary glands were harvested from female 5-week mice. Tissues were fixed in 4% paraformaldehyde and paraffin embedded, while tumors were fixed in 10% neutral buffered formalin and paraffin embedded. Following rehydration and heat-induced antigen retrieval for 20 minutes, sections were blocked for 1 hour in 10% normal goat serum and stained for LYVE-1 (1:50, R&D Systems #AF2125) and either CD206 (1:1,000, Abcam # ab64693) or F4/80 (Cell Signaling Technology, clone D2S9R) at 4°C overnight. The next day, sections were stained with biotinylated HABP (1:100, MilliporeSigma #385911) for 1 hour at room temperature. Sections were then incubated with secondary antibodies Streptavidin Alexa Fluor 647 (1:400, Thermo Fisher Scientific S32357), goat anti-rat Alexa Fluor 594 (1:400, Thermo Fisher Scientific A11012), and donkey anti-goat Alexa Fluor 488 (1:500, Thermo Fisher Scientific A11055) and sealed with DAPI ProLong Gold Antifade Mountant.

Additional slides were stained with hematoxylin and eosin. Slides were imaged using a Keyence BZ-X800 Series Microscope using the 20X objective and image stitching was done in proprietary Keyence software. Additional samples were stained with anti-phospho-Histone H3 (Cell Signaling Technology 9701) and DeadEnd fluorometric TUNEL System (Promega) and quantified at 40X magnification.

Alternatively, following antigen retrieval and blocking, sections were stained for CD8a (Cell Signaling Technology, clone D4W2Z) followed by secondary antibody donkey anti-Rabbit Alexa Fluor 488 (1:500, Invitrogen) and sealed with DAPI ProLong Gold Antifade Mountant. Slides were imaged and quantified using a Keyence BZ-X800 Series Microscope with the 40X objective.

### HA ELISA

To assess HA content in mammary tissue, #3 and #4 mammary glands were harvested from female 5-week and 15-week mice. EO771 tumors were harvested from *Csf1r^fl/fl^* or *Lyve1^Cre^Csf1r^fl/fl^* female mice at 1.5 cm^3^. Tissue was homogenized in RIPA buffer and centrifuged at 14,000 × *g*. Supernatant was collected, applied to an HA ELISA (Thermo Fisher Scientific #K1200), and HA content was normalized by tissue weight. Supernatant was also used to measure hyaluronidase function in a Hyaluronidase Activity ELISA (Echelon K-6000).

### Mammary Gland Isolation and qRT-PCR

Mammary glands were harvested from 15-week females and lymph nodes were removed. Tissue was homogenized in TriPure to extract RNA and cDNA was prepared using the qScript cDNA synthesis kit according to the manufacturers’ protocols. Gene expression was analyzed using the RT² Profiler PCR Array: Mouse Extracellular Matrix & Adhesion Molecules (Qiagen, PAMM-013ZA-2) with a Bio-Rad iQ5 system (*n* = 3). The 2^−ΔΔCt^ method was used to determine relative quantification of gene expression and normalized to five loading controls. Additional cDNA from the same samples was probed with primers specific for *Lyve1*, *Hyal1*, and *Hyal2* (27). The primer sequences are as follows: *Lyve-1* Forward- 5′-ACT TGC AGC TAT GGA TGG GT-3′, *Lyve-1* Reverse- 5′-GGA GTT AAC CCA GGT GTC GG-3′, *Hyal1* Forward- 5′-TGC TCA GAA AGT TTG GAG AAT GAA G-3′, *Hyal1* Reverse-5′-AAA GTC AGG AAG AGA GTA GAG ATG C-3′, *Hyal2* Forward-5′-TCT TCA CGC GTC CCA CAT AC-3′, *Hyal2* Reverse-5′-CAC TCT CAC CGA TGG TAG AGA TAA G-3′.

### Injection of EO771 and WNT-4226-65L Cells into the Mammary Fat Pad

The WNT-4226 cell line, provided by Dr. James Jackson (Tulane University, New Orleans, LA), was created from a spontaneous MMTV-*Wnt* tumor, as described by Shahbandi and colleagues (28, 29). To generate a cell line that reproducibly forms mammary tumor following orthotopic injection *in vivo*, the WNT-4226 line was orthotopically injected into the #4 mammary fat pad of female mice in a 50% Matrigel/PBS solution. Tumors were harvested, minced, and frozen in 10% DMSO in FBS. Tumor chunks were thawed and orthotopically implanted into the #4 mammary gland of female mice. Tumors were subsequently harvested and purified by anti-EpCAM microbeads (Milentyi Biotec #130-105-958) and cell lines were propagated *in vitro*, labeled WNT-4226-65L.

To induce tumor formation, 7.5 × 10^4^ EO771 cells or 1 × 10^6^ WNT-4226-65 L cells were resuspended in 50% Matrigel/PBS solution and orthotopically injected into the fourth mammary gland. Once palpable, tumors were measured using calipers every other day to determine growth rate and total tumor volume. Animals were humanely euthanized using CO_2_ once tumors reached endpoint (0.5 or 1.5 cm^3^). Tumors were harvested for flow cytometry, histologic analysis, immunofluorescence, or HA quantification.

### EO771 Lung Colonization Model

A total of 1 × 10^6^ EO771 tumor cells were injected via tail vein of *Csf1r^fl/fl^* or *Lyve1^Cre^Csf1r^fl/fl^* female mice. After 14 days, lungs were harvested, formalin fixed, and paraffin embedded. Lungs were stained by hematoxylin and eosin (H&E) and lung colonization was quantified as percent total area of the lung in ImageJ.

### *In Vitro* Induction of J774 Macrophages

To induce LYVE-1 expression *in vitro,* J774 cells were stimulated with 30 ng/mL *Escherichia coli*-derived mouse MCSF in base medium on day 0. On day 4, media was removed and cells were stimulated with 10 ng/mL *E. coli*-derived mouse IL4, 100 nmol/L dexamethasone in 75% EO771 conditioned media and 25% base media. Control wells received base media on day 0, and 25% base media and 75% DMEM on day 4. On day 7, media was removed and LYVE-1 (1:50, R&D Systems #AF2125) induction was quantified by flow cytometry. Flow cytometry was performed using an LSR Fortessa X-20 (BD Biosciences) and analyzed with FlowJo Software.

To analyze hyaluronidase content and function, LYVE-1 induced and control J774 macrophages were lysed in RIPA contained phosphatase and protease inhibitors. Hyaluronidase content was examined by Hyaluronidase ELISA (LS-Bio LS-F9648-1) and function was analyzed by hyaluronidase activity ELISA (Echelon K-6000).

### *In Vitro* HA Internalization

To evaluate HA internalization, 0.25 mg/mL TexasRed conjugated low molecular weight-hyaluronan (LMW-HA, 30 kDa, Echelon Biosciences) or TexasRed conjugated high molecular weight-hyaluronan (HMW-HA, 850 kDa, Echelon Biosciences) was added to wells with or without induction treatment for 45 minutes. Media was removed and cells were washed in PBS then scraped. To differentiate internalized HA compared with externally bound HA, cell surface proteins were cleaved with 0.5 mg/mL papain (Neta). Internalization was evaluated by flow cytometry. Flow cytometry was performed using an LSR Fortessa X-20 and analyzed with FlowJo Software. Additional samples were pretreated with a LYVE-1 blocking antibody or isotype control followed by culture with LMW-HA or HMW-HA and HA binding was assessed by flow cytometry.

### scRNA-seq and Analysis

EO771 tumors from *Csf1r^fl/fl^* or *Lyve1^Cre^Csf1r^fl/fl^* female mice were harvested at 1.5 cm^3^ (*n* = 2 per genotype) and dissociated via the method described above. Following red blood cell removal and blocking, cells were stained at room temperature with fixable viability dye (eBiosciences), CD45 (BD Biosciences, 30-F11), and respective hashtagging antibodies (TotalSeq A0301 anti-mouse Hashtag 1 or TotalSeq A0302 anti-mouse Hashtag 2). Cells were sorted with a BD FACSAria II cell sorter to enrich for CD45^+^ cells.

scRNA-seq libraries were constructed with the Chromium Single Cell 3′ Library and Reagent Kit v2 (10x Genomics) and the Chromium v2 Single Cell platform (10x Genomics). Libraries were sequenced at the University of Minnesota Genomics Center using one high-output run on a HiSeq 2500 instrument. Sequenced reads were assigned to gene expression libraries or HTO libraries, and mapped to the mouse genome (mm10), using cellranger v6.0.1 (10x Genomics) to produce cell-by-gene count matrices for further analysis using Seurat v4.3.0 (30, 31).

Preliminary clustering identified a subset of clusters with cells originating almost exclusively from the *Lyve1^Cre^Csf1r^fl/fl^* tumor in the second Chromium run. These four genes were identified as lymphocytes with high expression of ribosomal genes and were excluded from subsequent analysis. After removing these cells and reclustering, the remaining cells formed 17 distinct clusters.

Count matrices from cellranger were converted to Seurat objects using the function Read10X and filtered to include only cells flagged as singlets with <5% mitochondrial reads. RNA counts were processed in Seurat using the R function SCTransform with the percent of mitochondrial genes included in the “vars.to.regress” argument. Transformed counts were then passed to the R functions RunPCA, RunUMAP, and FindNeighbors using the first 30 principle components. Clusters were identified using the R function FindClusters with a clustering resolution of 0.6. Potential contaminant clusters were identified as clusters with >90% of cells originating from one of the four samples. Four clusters were identified as specific to the second *Lyve1^Cre^Csf1r^fl/fl^* tumor (“Tumor, Cre^+^, Rep2”) and all cells from all samples in those clusters were removed from subsequent analysis. Seurat AddModuleScore function was used to show expression of genes upregulated in LYVE-1^+^ cells in a previous study (18).

Gene set enrichment analyses (GSEA) were performed on lists of genes detected in at least 5% of cells in a comparison. Genes were ordered in the lists according to *P* value and direction of differential expression as determined using Seurat's FindAllMarkers. GSEA was performed using the GSEA function from ClusterProfiler v4.6.2 with a Benjamini–Hochberg FDR calculation (32). Mouse gene sets were restricted to lists with 30–500 genes from the MH (orthology-mapped hallmark gene sets), M2 (curated gene sets), and M5 (ontology gene sets) from the Molecular Signatures Database (MSigDB).

### Quantification and Statistical Analysis

Statistical analysis was performed using Student unpaired, two-tailed *t* test. Error bars represent SEM. Tumor growth sizes were compared by using two-way ANOVA with Tukey multiple comparison test on each timepoint. Significance is denoted as: *, *P <* 0.05; **, *P <* 0.01; ***, *P <* 0.001. Statistical details, including statistical tests used, number of animals, and precision measures, can be found in the figure legends of each figure. GraphPad Prism was used for statistical analysis calculations.

### Resource Availability

#### Lead Contact

Further information and requests for resources and reagents should be directed to and will be fulfilled by the lead contact, Kathryn L. Schwertfeger (schwe251@umn.edu)

### Data and Code Availability

- The data generated in this study are available from the lead contact upon request. The datasets used and analyzed for scRNA-seq experiments in the study are available in the NCBI Gene Expression Omnibus database under the accession number GSE241469.
- Original code is available at https://zenodo.org/record/8322142.
- Any additional information required to reanalyze the data reported in this article is available from the lead contact upon request.

## Results

### LYVE-1^+^ Macrophages Localize Near Stromal HA in the Nulliparous Mouse Mammary Gland and are Associated with ECM Regulation

In previously published studies, we have shown that depletion of macrophages using a colony-stimulating factor 1 receptor (CSF1R) inhibitor led to accumulation of HA within the mammary gland stroma (18). However, because CSF1R inhibition led to the depletion of both LYVE-1^+^ and LYVE-1^−^ macrophages, these studies did not define the specific phenotypic subset of macrophages that contributes to HA modulation. LYVE-1 is known to contribute to HA internalization and degradation by LECs and has recently been found to promote HA internalization by macrophages (12, 33). Therefore, we hypothesized that LYVE-1–expressing macrophages contribute to HA organization in the mammary gland stroma. To address this hypothesis, we utilized a genetic model of LYVE-1^+^ macrophage depletion in which *Lyve1-Cre* mice were crossed with *Csf1r*-floxed (*Csf1r^fl/fl^*) mice to generate *Lyve1^Cre^Csf1r^fl/fl^* mice. While this model has been used by others to study the functional contributions of LYVE-1^+^ macrophages to the maintenance of arterial tone (13), it has not previously been used to assess LYVE-1^+^ macrophage function in cancer models. Because CSF1R is vital for macrophage survival, all cells that express *Lyve1* will delete the *Csf1r* gene, resulting in depletion of LYVE-1^+^ macrophages. Other cell types that express LYVE-1 include LECs, megakaryocytes, and platelets, which do not express CSF1R (34).

**FIGURE 1:**
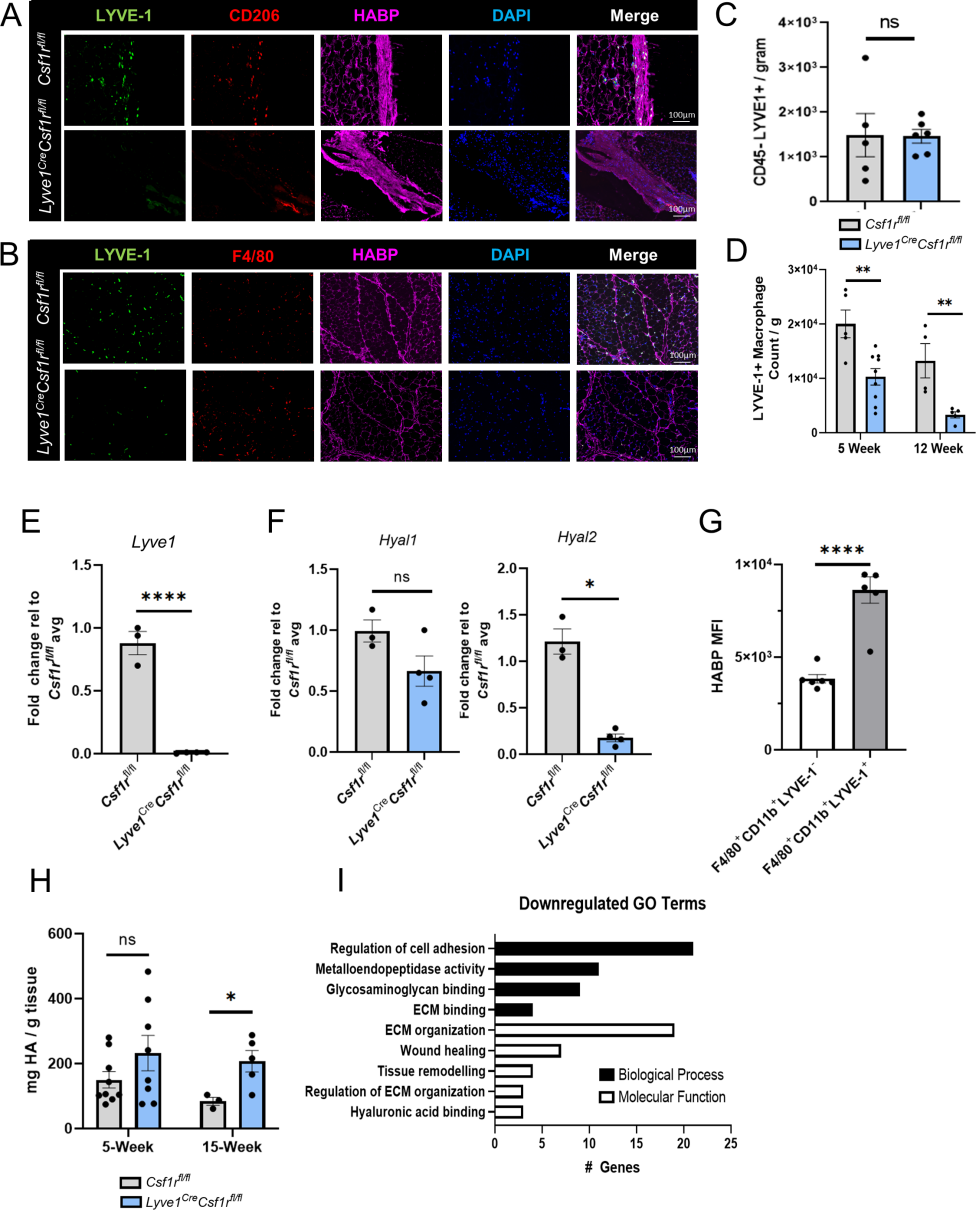
LYVE-1^+^ macrophages localize near stromal HA in the nulliparous mouse mammary gland and are associated with ECM regulation. **A,** Representative images from mammary gland capsular regions of 5-week *Csf1r^fl/fl^* (top) and *Lyve1^Cre^Csf1r^fl/fl^* (bottom) mice immunostained for LYVE-1 (green), CD206 (red), HABP (magenta), and DAPI (blue; *n* = 4 per genotype). **B,** Representative images from mammary gland stroma regions of 5-week *Csf1r^fl/fl^* (top) and *Lyve1^Cre^Csf1r^fl/fl^* (bottom) mice immunostained for LYVE-1 (green), F4/80 (red), HABP (magenta), and DAPI (blue; *n* = 4 per genotype). **C,** Mammary glands from 5-week *Csf1r^fl/fl^* (gray) and *Lyve1^Cre^Csf1r^fl/fl^* (blue) female mice were assessed for CD45^−^LYVE-1^+^ frequency by flow cytometry. **D,** Mammary glands from 5-week *Csf1r^fl/fl^* (gray) and *Lyve1^Cre^Csf1r^fl/fl^* (blue) female mice were assessed for CD45^+^F4/80^+^CD11b^+^ LYVE-1^+^ macrophage count by flow cytometry. Fold change of *Lyve1* (**E**), *Hyal1*, and *Hyal2* (**F**) RNA expression from 15-week *Csf1r^fl/fl^* (gray) and *Lyve1^Cre^Csf1r^fl/fl^* (blue) female mice. **G,** MFI of HABP in mammary glands assessing CD45^+^F4/80^+^CD11b^+^LYVE-1^−^ and CD45^+^F4/80^+^CD11b^+^LYVE-1^+^ macrophages. **H,** Mammary gland from 5-week and 15-week *Csf1r^fl/fl^* (gray) and *Lyve1^Cre^Csf1r^fl/fl^* (blue) female mice assessed for HA by ELISA and normalized by weight. **I,** Female mammary glands assessed by ECM qRT-PCR array with differentially downregulated genes associated with biological process Gene Ontology (GO) terms (black) and molecular function GO terms (white; *n* = 3 per genotype). *, *P <* 0.05; **, *P <* 0.01; ****, *P <* 0.0001, using Student *t* test. Scale bars, 100 µm. Each dot represents one mouse.

We have previously determined that LYVE-1^+^ macrophages selectively associate with HA-containing regions of connective tissue in the mammary gland stroma (18). Thus, to validate the successful depletion of LYVE-1^+^ macrophages from the mammary glands, we assessed the localization of LYVE-1^+^ macrophages to HA-enriched stromal regions. Immunofluorescence staining demonstrated LYVE-1 colocalization with the tissue-resident macrophage marker CD206 and HABP in the capsular region of mammary glands from 5-week-old nulliparous female mice (Fig. 1A). In addition, LYVE-1 colocalized with the pan-macrophage marker F4/80 and HABP in the stromal compartment of mammary glands (Fig. 1B). Further supporting the successful depletion of LYVE-1^+^ macrophages in this model, LYVE-1^+^ macrophages were reduced within the HA-associated compartments in mammary glands from *Lyve1^Cre^Csf1r^fl/fl^* mice (Fig. 1A and B). These data demonstrate effective depletion of LYVE-1^+^ macrophages in our genetic model and distinct colocalization of LYVE-1^+^ macrophages with HA in mammary stroma.

To quantify the extent of depletion of LYVE-1^+^ macrophages in this model, mammary glands were isolated from 5-week female *Lyve1^Cre^Csf1r^fl/fl^* and *Csf1r^fl/fl^* mice and analyzed by flow cytometry. No changes were observed in CD45^−^LYVE-1^+^ cell counts, indicating that nonimmune LYVE-1^+^ cells, such as LECs, were retained in the mammary glands of these mice (Fig. 1C; Supplementary Fig. S1A). Additional studies were performed to quantify total and LYVE-1^+^ macrophage populations in mammary glands from nulliparous female *Lyve1^Cre^Csf1r^fl/fl^* and control *Csf1r^fl/fl^* mice using flow cytometry. While there were downward trends in total macrophage counts in mammary glands from *Lyve1^Cre^Csf1r^fl/fl^* and *Csf1r^fl/fl^* mice at both 5 and 12 weeks of age (Supplementary Fig. S1B), these differences did not reach statistical significance. However, a significant reduction in the number of LYVE-1^+^ macrophages was observed in mammary glands from *Lyve1^Cre^Csf1r^fl/fl^* mice compared with *Csf1r^fl/fl^* mice at both timepoints (Fig. 1D; Supplementary Fig. S2). These data indicate that LYVE-1^+^ macrophages are found in association with HA and are effectively depleted in the normal mammary gland.

Because we have previously found LYVE-1^+^ macrophages localize to HA-enriched stromal regions of mammary glands (18), initial studies were performed to determine the role of LYVE-1^+^ macrophages in orchestrating general HA-associated remodeling in the whole mammary gland. RNA was extracted from whole mammary glands, including their fat pads, from female *Lyve1^Cre^Csf1r^fl/fl^* and *Csf1r^fl/fl^* mice at 15 weeks of age. As expected, there was a significant reduction in *Lyve1* expression in whole mammary glands from *Lyve1^Cre^Csf1r^fl/fl^* mice compared with control mice (Fig. 1E). To investigate HA regulation in mammary glands, expression of the HA degrading enzymes hyaluronidase (HYAL) 1 and 2 were analyzed by qRT-PCR. While there was not a significant difference in *Hyal1* expression, *Hyal2* expression was downregulated in mammary glands from *Lyve1^Cre^Csf1r^fl/fl^* mice compared with controls, suggesting that depletion of LYVE-1^+^ macrophages correlates with reduced expression of HA degrading enzymes in the mammary gland (Fig. 1F). To determine whether LYVE-1^+^ macrophages are capable of binding HA *in vivo*, flow cytometry was performed on macrophages isolated from mammary glands from wild-type C57BL/6 mice. Quantification of HABP mean fluorescent intensity (MFI) demonstrated that more HA was bound to LYVE-1^+^ macrophages compared with LYVE-1^−^ macrophages (Fig. 1G). Finally, to determine whether depletion of LYVE-1^+^ macrophages alters HA accumulation in the mammary gland, an ELISA was performed to quantify HA levels in mammary glands from 5- and 15-week-old nulliparous female mice. While no differences in HA levels were observed in mammary glands from 5-week-old mice, there was a significant increase in HA in *Lyve1^Cre^Csf1r^fl/fl^* mammary glands from 15-week-old mice, which correlates with the reduction in *Hyal2* expression in mammary glands from these mice (Fig. 1H). These data indicate that LYVE-1^+^ macrophages bind HA and that their presence correlates with the expression of genes associated with HA degradation, suggesting a role for LYVE-1^+^ macrophages in HA regulation in the mammary gland.

To determine whether LYVE-1^+^ macrophages have effects on other aspects of ECM remodeling, expression levels of additional genes associated with ECM adhesion and remodeling were analyzed using a targeted qRT-PCR array. Analysis of RNA isolated from whole mammary gland tissue demonstrated that LYVE-1^+^ macrophage depletion correlated with reduced expression of metalloproteases, ECM genes such as *Fn1*, integrins, cell adhesion molecules, and collagens such as *Col1a1* and *Col2a1* (Supplementary Fig. S3A; Fig. 1I). Despite the changes in collagen and *Fn1* genes, no changes in pro-collagen 1 alpha 1 (Col1a1) or fibronectin were identified in female mammary glands by ELISA. These findings suggest that in contrast to HA, depletion of LYVE-1^+^ macrophages may not significantly impact the levels of other ECM components in mammary tissue (Supplementary Fig. S3B and S3C).

Because changes in stromal HA in the mammary glands of pubertal mice were observed with LYVE-1^+^ macrophage depletion, further studies were performed to determine whether LYVE-1^+^ macrophage depletion affects ductal elongation in the mammary gland. Mammary glands were harvested from *Lyve1^Cre^Csf1r^fl/fl^* and *Csf1r^fl/fl^* mice at 7 weeks of age and whole mounts were analyzed for ductal elongation (Supplementary Fig. S3D). We did not observe significant changes in ductal elongation at this timepoint, suggesting that the changes in stromal HA are not sufficient to impact ductal elongation in the mammary gland. These findings indicate that depletion of LYVE-1^+^ macrophages in the mammary gland correlates with altered expression of tissue remodeling factors and increased HA accumulation, but that these changes do not directly impact ductal elongation.

### Identification and Localization of LYVE-1^+^ Macrophages in Mammary Tumors

**FIGURE 2:**
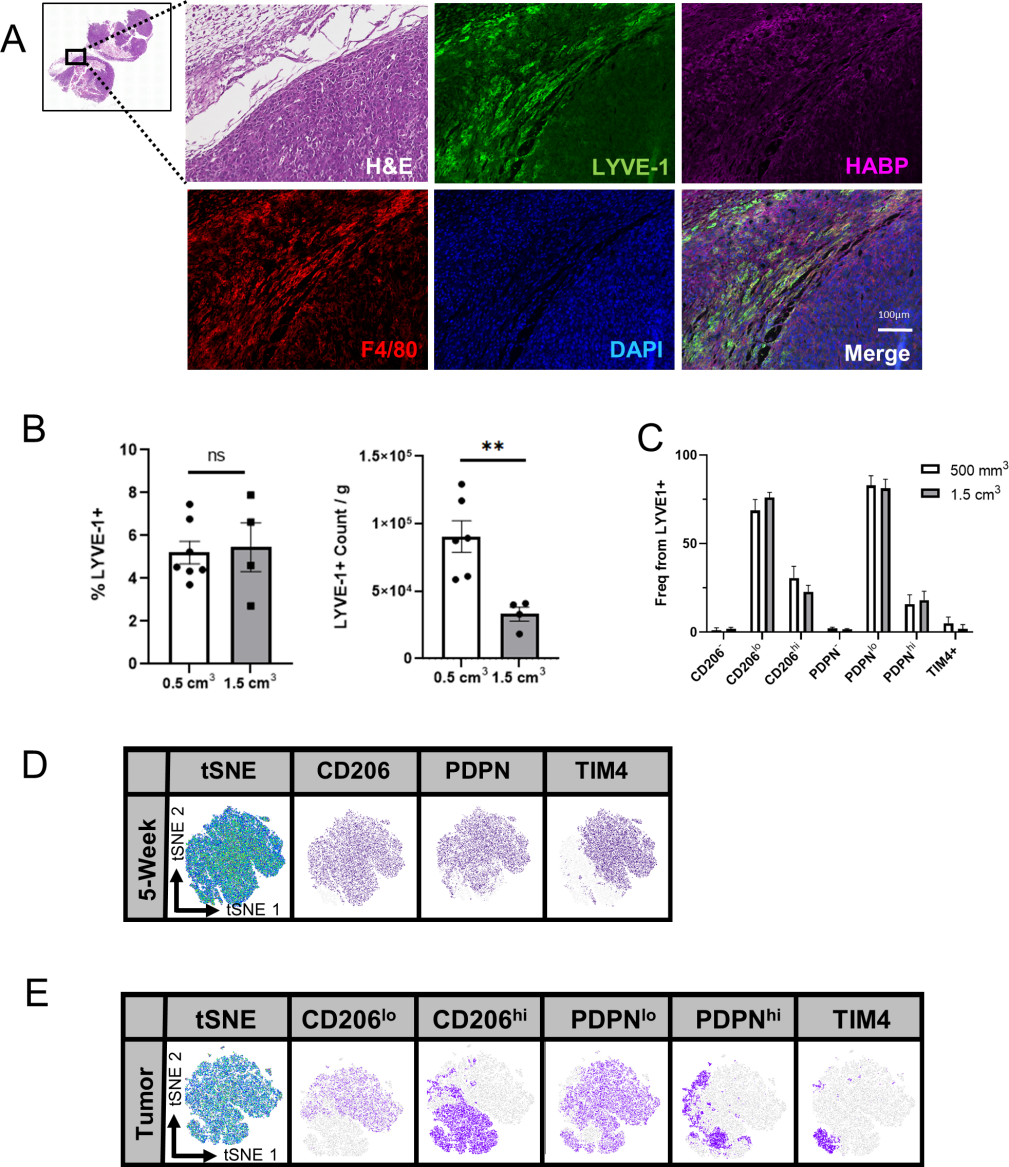
Identification and localization of LYVE-1^+^ macrophages in mammary tumors. **A,** Representative images from formalin-fixed and paraffin-embedded (FFPE) sections of EO771 tumors from *Csf1r^fl/fl^* mice stained by H&E, and immunostained for LYVE-1 (green), HABP (magenta), F4/80 (red), and DAPI (blue; *n* = 5). CD45^+^F4/80^+^CD11b^+^LYVE-1^+^ macrophage count, CD45^+^F4/80^+^CD11b^+^LYVE-1^+^ macrophage count normalized to tumor weight (**B**), and frequency of CD206, PDPN, and TIM4 from CD45^+^F4/80^+^CD11b^+^LYVE-1^+^ macrophage subset in EO771 tumors from C57BL/6 mice (**C**). FlowJo generated tSNE plots from CD45^+^F4/80^+^CD11b^+^LYVE-1^+^ subset in 5-week female mammary glands (**D**), and EO771 C57BL/6 tumors (**E**). **, *P <* 0.01. Scale bars, 100 µm. Each dot represents one mouse.

In addition to providing structural support for normal tissues, HA is also a prominent structural component found in tumors (35). Thus, further studies focused on determining the interactions between LYVE-1^+^ macrophages and HA in mammary tumors. Using the 4T1 mammary tumor model, we have previously shown that LYVE-1^+^ macrophages localize to the peritumoral HA-containing stroma (18). Because the *Lyve1^Cre^Csf1r^fl/fl^* model is maintained on the C57BL/6 background, we performed further studies using the C57BL/6-derived EO771 mammary tumor model (36). Initial studies assessed the localization of LYVE-1^+^ macrophages within the HA-containing peritumoral stroma. Similar to the 4T1 model, LYVE-1^+^ and F4/80^+^ macrophages were found to be associated with the tumor margin (Fig. 2A). Furthermore, LYVE-1^+^ and F4/80^+^ macrophages were found to localize in close proximity to HA dense tumor regions within the peritumoral stroma (Fig. 2A).

On the basis of our findings that LYVE-1^+^ macrophages are associated with a phenotype linked with ECM remodeling, which is important for tumor growth and progression, we sought to examine the presence and function of LYVE-1^+^ macrophages in tumors. A total of 7.5 × 10^4^ EO771 mammary tumor cells were orthotopically implanted into the mammary fat pads of recipient C57BL/6 mice and mammary tumors were harvested at sizes of 0.5 and 1.5 cm^3^, representing early- and late-stage tumors, and LYVE-1^+^ macrophages were evaluated by flow cytometry (Fig. 2B). LYVE-1^+^ macrophages were considered CD45^+^, F4/80^+^, CD11b^+^ as shown in representative flow plots (Supplementary Fig. S4). No changes in LYVE-1^+^ macrophage frequency were found between tumor sizes; however, a reduction in LYVE-1^+^ macrophage count was found in the 1.5 cm^3^ tumors (Fig. 2B).

Other studies have shown that LYVE-1^+^ macrophages may express additional cell surface markers such as CD206, PDPN, and TIM4 (14, 37–38). Thus, further investigation of the overlap between these markers and LYVE-1 was performed in both the normal mammary gland and in mammary tumors to evaluate the heterogeneity of tumor-associated LYVE-1^+^ macrophages. Heterogeneity was confirmed with flow cytometric analysis of both normal mammary gland and EO771 tumors, with varying expression of CD206, PDPN, and TIM4 observed (Fig. 2C–E). Expression of CD206, PDPN, and TIM4 did not change as tumors grew, suggesting that their expression was not associated with tumor stage. Using FlowJo to generate tSNE plots, CD206 and PDPN were largely coexpressed throughout the population in normal mammary glands (Fig. 2D; representative flow plots in Supplementary Fig. S4). TIM4 expression, typically associated with tissue-resident macrophages (37, 39), was found in approximately half of mammary gland LYVE-1^+^ macrophages. Unlike macrophages in the mammary gland, tumor-associated macrophages expressed CD206 and PDPN with greater variability, producing CD206^−^, CD206^lo^, and CD206^hi^ populations, and PDPN^−^, PDPN^lo^, and PDPN^hi^ populations, respectively (Fig. 2E). In contrast to the high levels of TIM4 expression in LYVE-1^+^ macrophages in the normal mammary gland, TIM4 expression in LYVE-1^+^ macrophages from tumors was limited, suggesting that there may be different origins between mammary gland and tumor LYVE-1^+^ macrophages. The small population of TIM4^+^ cells was found with the CD206^hi^ population, while the PDPN^hi^ population largely aligned with the CD206^hi^ group. These data demonstrate that LYVE-1^+^ macrophages exhibit phenotypic diversity in a context-dependent manner.

### Genetic Depletion of LYVE-1^+^ Macrophages Delays Mammary Tumor Growth

**FIGURE 3:**
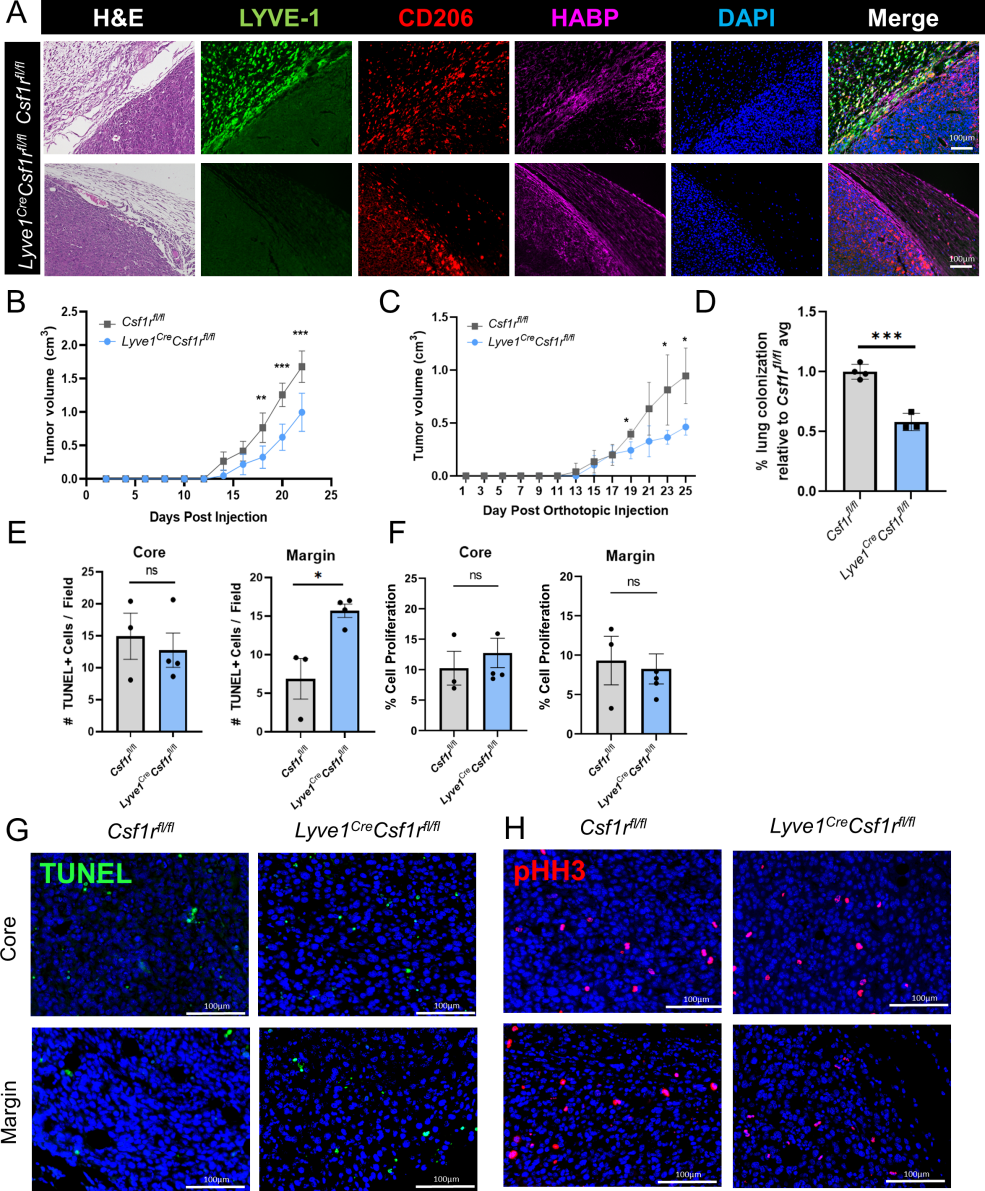
Genetic depletion of LYVE-1^+^ macrophages delays mammary tumor growth. **A,** Representative images from FFPE sections of EO771 tumors from *Csf1r^fl/fl^* (top) and *Lyve1^Cre^Csf1r^fl/fl^* (bottom) mice stained by H&E, and immunostained for LYVE-1 (green), HABP (magenta), CD206 (red), and DAPI (blue; *n* = 5). **B,** EO771 tumor growth curve from *Csf1r^fl/fl^* (gray, *n* = 4) and *Lyve1^Cre^Csf1r^fl/fl^* (blue, *n* = 4) mice, cutoff when the first mouse reaches endpoint. **C,** WNT-4226-65 L tumor growth curve from *Csf1r^fl/fl^* (gray, *n* = 4) and *Lyve1^Cre^Csf1r^fl/fl^* (blue, *n* = 4) mice, cutoff when the first mouse reaches endpoint. **D,** Lung colonization of EO771 tumor cells measured by H&E staining. Colonization is quantified as percent colonization per lung by area, relative to experimental *Csf1r^fl/fl^* average. Quantification of TUNEL (**E**) and pHH3 (**F**) staining of EO771 tumors from *Csf1r^fl/fl^* (gray) and *Lyve1^Cre^Csf1r^fl/fl^* (blue) mice from the core and margin. Representative images of TUNEL (**G**) and pHH3 (**H**) staining of EO771 tumors from *Csf1r^fl/fl^* and *Lyve1^Cre^Csf1r^fl/fl^* mice from the core and margin. *, *P <* 0.05; **, *P <* 0.01. Scale bars, 100 µm. Each dot represents one mouse.

Further studies were performed to evaluate the contributions of LYVE-1^+^ macrophages to tumor growth using the *Lyve1^Cre^Csf1r^fl/fl^* and *Csf1r^fl/fl^* mice. A total of 7.5 × 10^4^ EO771 tumor cells were orthotopically implanted into mammary fat pads of *Lyve1^Cre^Csf1r^fl/fl^* and *Csf1r^fl/fl^* mice and tumors were grown to 1.5 cm^3^. EO771 tumors from *Lyve1^Cre^Csf1r^fl/fl^* and *Csf1r^fl/fl^* were stained by H&E and immunostained with LYVE-1, CD206, and HABP (Fig. 3A). CD206^+^ macrophages were found in both the tumor parenchyma and the peritumoral stroma, while LYVE-1^+^ macrophages were primarily localized in the tumor stroma. In *Lyve1^Cre^Csf1r^fl/fl^* mice, LYVE-1^+^ and CD206^+^ macrophages were effectively depleted, while CD206^+^ macrophages in the parenchyma were retained (Fig. 3A). This suggests the presence of a distinct peritumoral CD206^+^LYVE-1^+^ macrophage subset, independent from the parenchymal CD206^+^LYVE-1^−^ subset.

We found that EO771 tumors grown in *Lyve1^Cre^Csf1r^fl/fl^* mice showed a reduction in the rate of tumor growth compared with tumors in *Csf1r^fl/^^fl^* mice (Fig. 3B; Supplementary Fig. S5A and S5B). These findings were validated in a secondary model using the WNT-4226-65 L mammary tumor cell line, a cell line that was generated from a spontaneous MMTV-*Wnt1* tumor (28, 29), which we then subsequently passaged *in vivo* to generate a cell line that reproducibly forms mammary tumors in mice. A total of 1 × 10^6^ WNT-4226-65 L cells were orthotopically implanted into mammary fat pads of *Lyve1^Cre^Csf1r^fl/fl^* and *Csf1r^fl/fl^* mice and tumors were grown to 1.5 cm^3^ (Fig. 3C; Supplementary Fig. S5C and S5D). As an additional control, tumor growth was measured in *Lyve1^Cre/Cre^Csf1r^wt/wt^* mice and no change was found compared with the *Csf1r^fl/fl^* mice (Supplementary Fig. S5E). These results demonstrate that the reduction in tumor growth observed in *Lyve1^Cre^Csf1r^fl/fl^* mice is due to the loss of LYVE-1^+^ macrophages and not a result of Cre recombinase expression.

Because metastasis is the leading cause of cancer-related death, we next examined the contributions of LYVE-1^+^ macrophages to metastasis in the lung. Because we have not found robust metastasis of EO771 cells following orthotopic transplantation, a lung colonization model was used. LYVE-1^+^ macrophage depletion led to a reduction in growth of tumor cells in the lung following tail vein injection, indicating that LYVE-1^+^ macrophages support lung colonization (Fig. 3D).

Because of the observed reduction in tumor growth, further studies were performed to assess the effects of LYVE-1^+^ macrophage depletion on proliferation and apoptosis in the tumor. To investigate tumor apoptosis, TUNEL staining was performed on tumors derived from *Lyve1^Cre^Csf1r^fl/fl^* and *Csf1r^fl/fl^* mice. The tumor margin was defined as the region within 5 mm of the tumor edge, while the tumor core was defined as the region > 5 mm from the tumor edge. Quantification of apoptosis revealed that while no changes were observed in apoptosis in the tumor core, increased apoptosis was observed in *Lyve1^Cre^Csf1r^fl/fl^* mice compared with *Csf1r^fl/fl^* mice at the tumor margin (Fig. 3E and G). Immunofluorescent staining for phospho-Histone H3 identified no changes in proliferation in *Lyve1^Cre^Csf1r^fl/fl^* compared with *Csf1r^fl/fl^* mice at either the core or margin (Fig. 3F and H). These data indicate that the loss of LYVE-1^+^ macrophages correlates with increased tumor cell death at the margin, which is in proximity to peritumoral LYVE-1^+^ macrophages.

### LYVE-1 Expression Correlates with HA Internalization

**FIGURE 4:**
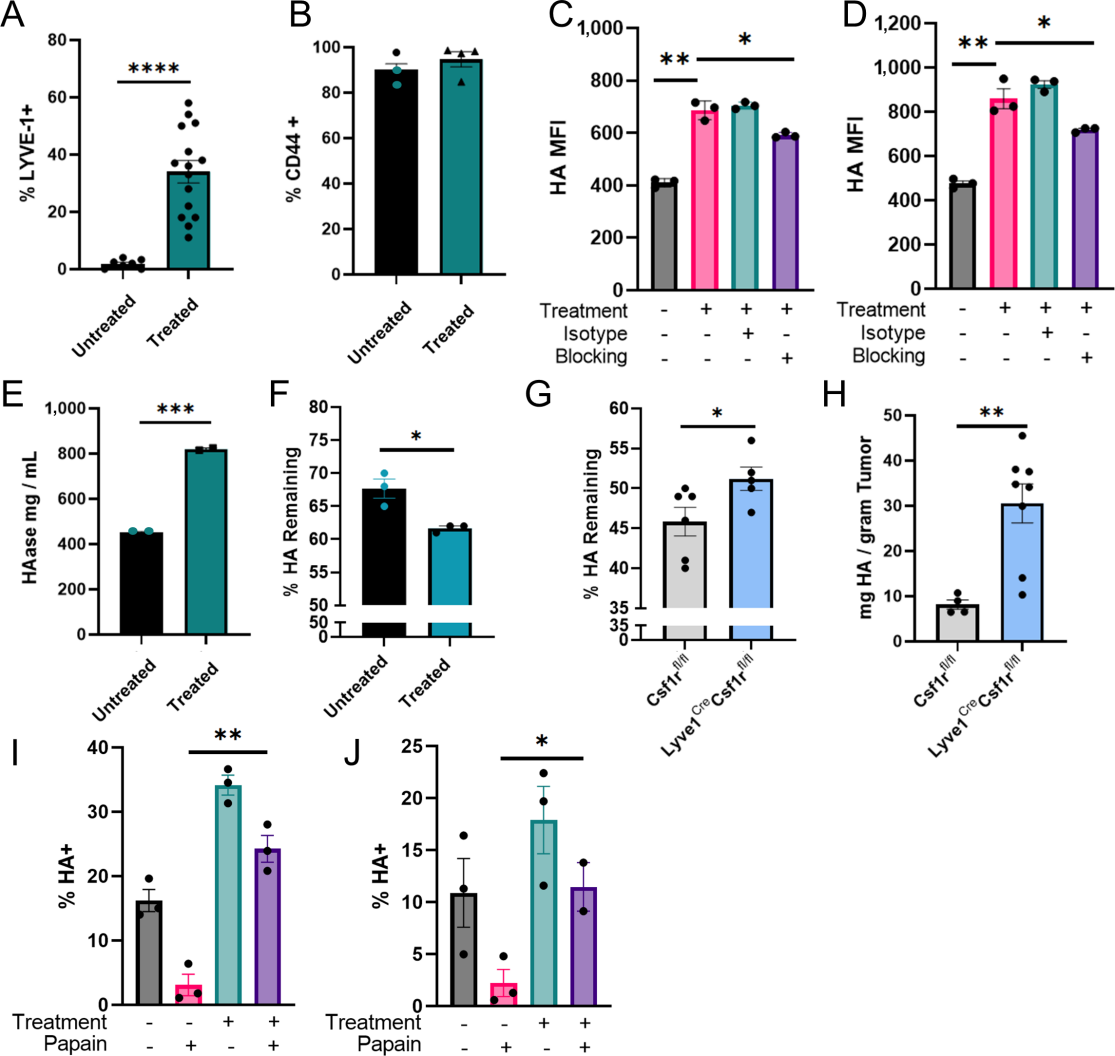
LYVE-1 expression correlates with HA internalization. J774 macrophages untreated or treated with MCSF, IL4, dexamethasone, and EO771 conditioned media assessed for LYVE-1 expression (**A**) and CD44 expression (**B**) by flow cytometry. J774 macrophages untreated or treated with MCSF, IL4, dexamethasone, and EO771 conditioned media and pretreated with an isotype control or LYVE-1 blocking antibody followed by incubation with LMW-HA (**C**) or HMW-HA (**D**) and assessed by flow cytometry. Untreated and treated J774 macrophages assayed for hyaluronidase protein by ELISA (**E**) and hyaluronidase activity assay (**F**). **G,** Tumor lysates from *Csf1r^fl/fl^* and *Lyve^Cre^ Csf1r^fl/fl^* mice assayed by HA Activity ELISA (*n* = 5). **H,** EO771 tumors from *Csf1r^fl/fl^* (gray) and *Lyve^Cre^ Csf1r^fl/fl^* (blue) mice assessed for HA by ELISA and normalized by weight. J774 macrophages untreated or treated and incubated with LMW-HA (**I**) or HMW-HA (**J**) and assessed for HA internalization by flow cytometry. *, *P <* 0.05; **, *P <* 0.01; ***, *P <* 0.001; ****, *P <* 0.0001. Each dot represents one replicate.

LYVE-1^+^ macrophages are linked to glycosaminoglycan binding and their depletion correlates with increased HA levels in the mammary gland (Fig. 1H), suggesting that LYVE-1^+^ macrophages may contribute to HA degradation. Thus, further studies were performed to determine whether tumor-associated LYVE-1^+^ macrophages modulate HA in the tumor microenvironment. While LYVE-1 on LECs and macrophages has been shown to internalize HA prior to degradation by hyaluronidases, this function has not previously been described in the context of cancer (12, 33). Therefore, we examined macrophage-mediated HA internalization using an *in vitro* cell culture system. Because J774 macrophages express low levels of LYVE-1, LYVE-1 expression was induced using a combination of dexamethasone, MCSF, IL4, and EO771-derived conditioned media, a method previously described by Dollt and colleagues (40). Subsequently, expression of LYVE-1 was assessed by flow cytometry. A significant increase in LYVE-1 expression was observed upon treatment of J774 cells with this combination of factors (Fig. 4A). Expression of CD44, another key HA binding cell surface protein, was not altered upon treatment with these factors (Fig. 4B).

To validate the dependence of HA binding on LYVE-1, J774 cells treated with or without MCSF, IL4, and EO771-derived conditioned media were pretreated with a LYVE-1 blocking antibody or isotype control prior to incubation with fluorescently-labeled LMW-HA (30 kDa) or HMW-HA (850 kDa) and analyzed by flow cytometry. A reduction in HA binding was observed in samples that were treated with the LYVE-1 blocking antibody, suggesting that LYVE-1 functionally contributes to the ability of macrophages to bind HA (Fig. 4C and D).

Further studies were performed to examine hyaluronidase expression and function following treatment of J774 cells with these factors. We found that higher levels of LYVE-1 expression correlate with increased levels of hyaluronidase protein as determined by ELISA (Fig. 4E). Furthermore, macrophages with higher levels of LYVE-1 expression also demonstrate an enhanced ability to fragment HA in an HA activity ELISA (Fig. 4F). These findings suggest a correlation between LYVE-1 expression on macrophages and enhanced ability to degrade HA. These findings were validated *in vivo,* as tumor lysates from *Lyve1^Cre^Csf1r^fl/fl^* mice had diminished capacity to degrade HA compared with *Csf1r^fl/f^* control mice (Fig. 4G). Furthermore, tumors harvested from *Lyve1^Cre^Csf1r^fl/fl^* mice were associated with higher levels of HA than tumors harvested from control mice, suggesting that similar to the normal mammary gland, LYVE-1^+^ macrophages also contribute to HA modulation in mammary tumors (Fig. 4H).

HA internalization has been linked to HA degradation in other cell types (33). To assess the ability of LYVE-1–expressing J774 macrophages to internalize HA, J774 cells were treated with or without MCSF, IL4, and EO771-derived conditioned media and incubated with fluorescently-labeled LMW-HA or HMW-HA. The cells were then subjected to papain treatment, which cleaves extracellular proteins. This step allows for the detection of internalized HA rather than externally bound HA (12). The results from these experiments indicate that higher levels of LYVE-1 expression correlate with increased internalization of both LMW- and HMW-HA, indicating internalization of HA is irrespective of size (Fig. 4I and J). Together, these findings indicate that LYVE-1 expression levels on macrophages correlate with an enhanced ability to internalize and degrade HA, which is consistent with the finding that depletion of LYVE-1^+^ macrophages results in increased HA accumulation *in vivo*.

### Identification of Macrophage Subsets in the Tumor Microenvironment by scRNA-seq

The LYVE-1^+^ macrophage depletion model provides the unique opportunity to determine how loss of LYVE-1^+^ macrophages affects tissue remodeling and other phenotypes within the remaining macrophages. To assess this, scRNA-seq was performed on CD45^+^ immune cells isolated from EO771-derived mammary tumors from *Lyve1^Cre^Csf1r^fl/fl^* and *Csf1r^fl/f^* mice. A total of 7.5 × 10^4^ EO771 cells were orthotopically implanted into mammary fat pads of female mice and tumors were harvested at a size of 1.5 cm^3^ for flow cytometry. Viable CD45^+^ cells were sorted from two tumors per genotype and applied to the 10X Chromium platform using TotalSeq hashtag oligos to distinguish samples and identify gel beads containing cells from more than one sample. Clustering revealed 18 unique populations; however, one cluster was removed because of high expression of ribosomal genes.

**FIGURE 5:**
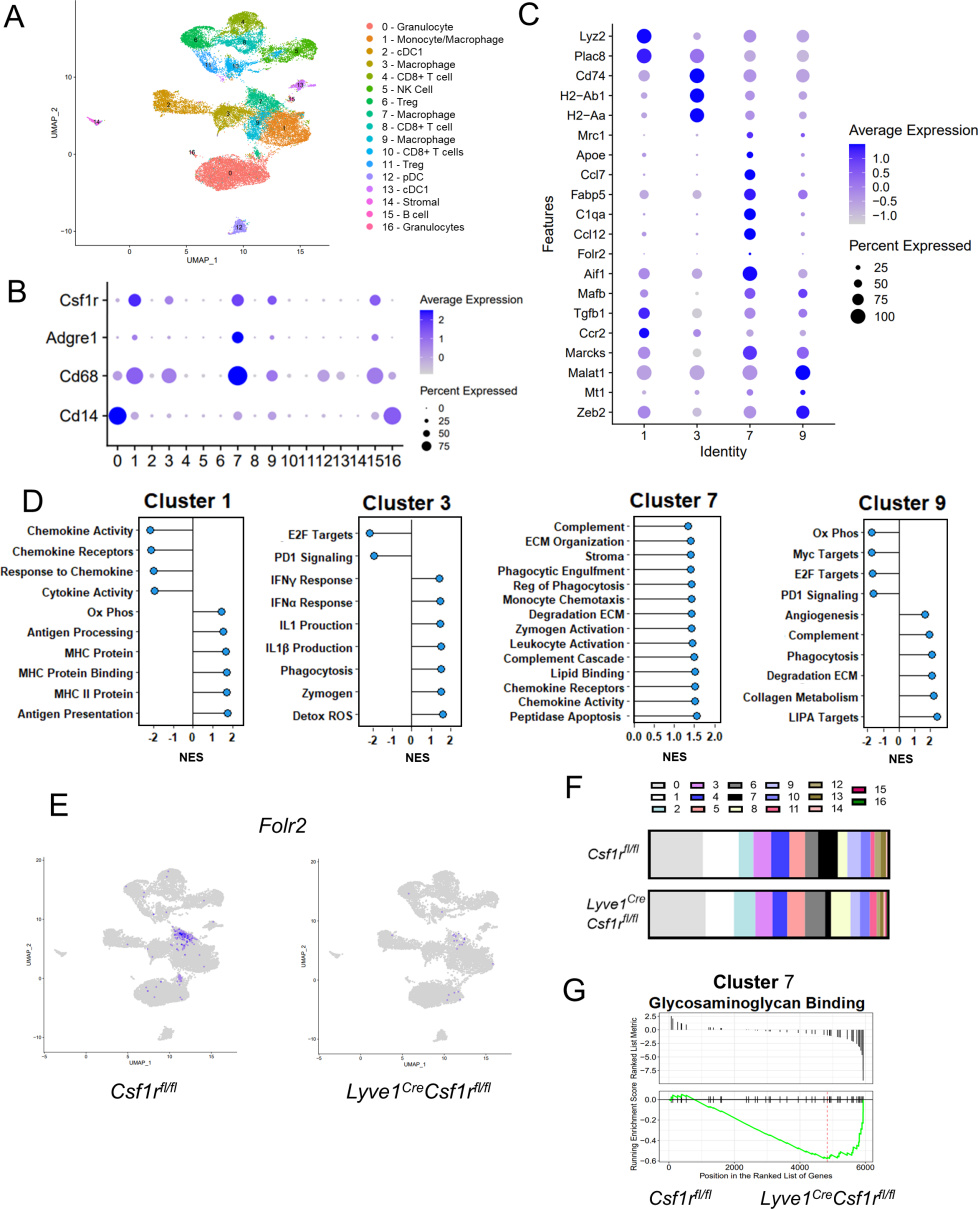
scRNA-seq identifies macrophage subsets in the tumor microenvironment. **A,** UMAP projection of 17 distinct clusters from the total population of CD45^+^ cells from EO771 tumors from *Csf1r^fl/fl^* and *Lyve1^Cre^Csf1r^fl/fl^* mice (*n* = 2 per genotype) with cell-specific labels. **B,** Dot plot of macrophage-associated genes from cells derived from *Csf1r^fl/fl^* tumors. **C,** Dot plot of enriched genes in macrophage clusters. **D,** Intercluster GSEA from *Csf1r^fl/fl^* macrophage clusters [normalized enrichment scale (NES) adjusted *P*-value < 0.05] with detailed gene set names in Supplementary Table S2. **E,** Feature plot of *Folr2* from tumors from *Csf1r^fl/fl^* and *Lyve1^Cre^Csf1r^fl/fl^* mice. **F,** Average cell counts per cluster from tumors from *Csf1r^fl/fl^* and *Lyve1^Cre^Csf1r^fl/fl^* mice. **G,** Waterfall plots of relative enrichment in cluster 7 *Lyve1^Cre^Csf1r^fl/fl^* relative to *Csf1r^fl/fl^* of genes associated with glycosaminoglycan binding.

Immune populations were identified in 16 of the 17 clusters, with cluster 14 considered contaminating stromal cells (Fig. 5A). Lineage markers *Cd8a, Foxp3, Nkg7, Csf1r, Cd19*, and *S100a9* were used to identify CD8^+^ T cells, regulatory T cells, NK cells, macrophages, B cells, and granulocytes, respectively (Supplementary Fig. S6). Cluster 0 was enriched in *S100a8* and *S100A9*, consistent with granulocytes. Cluster 1 was associated with high levels of *Ly6c2* and *Lyz2*, consistent with monocytes. Clusters 4, 6, 8, 10, and 11 expressed high levels of *Cd3e*, consistent with T cells. Within these T-cell clusters, cluster 4, 8, and 10 expressed *Cd8a* while cluster 6 and 11 expressed *Cd4* and *Foxp3*, representing CD8 and regulatory T cells, respectively. The top genes for each cluster are identified in a heat map found in Supplementary Fig. S7 and Supplementary Table S1.

To define macrophage clusters, we assessed expression of *Csf1r, Adgre1*, *Cd68*, and *Cd14* genes as we have previously described in Ibrahim and colleagues (Fig. 5B; Supplementary Fig. S6; ref. 41). Using these markers and excluding other lineage markers, macrophages were identified in clusters 1, 3, 7, and 9. Of the four macrophage clusters, cluster 1 exhibited differential expression of *Ccr2* and *Ly6c2*, consistent with a monocyte-derived macrophage population (Fig. 5C). Cluster 3 was associated with high levels of expression of genes associated with antigen presentation including *Cd74*, *H2-Ab1*, and *H2-Aa* (42). Cluster 7 was associated with lipid-associated macrophage genes (*Fabp5*, *Apoe*; ref. 43) and complement genes (*C1qa*, *C1qb*). C1q is a complement protein produced by tissue-resident macrophages, and macrophages that coexpress both *C1qa* and *Apoe* represent a tumor-supporting, prometastatic population (44, 45). Finally, cluster 9 was comprised of a macrophage population marked by *Malat1*, an M2-like tumor-supporting long noncoding RNA (Fig. 5C; refs. 46–48).

Differential gene expression data are supported by GSEA, comparing enriched gene sets between clusters of *Csf1r^fl/fl^* tumors (Fig. 5D; Supplementary Table S2). Cluster 1 is negatively enriched for chemokine gene sets and is positively enriched for antigen presentation. Cluster 3 is associated with inflammatory gene sets such as IFN response and IL1β production. Notably, the gene expression of cluster 7 is associated with tissue remodeling, phagocytosis, complement, and zymogen activation. Finally, the gene expression of cluster 9 is associated with collagen metabolism, phagocytosis, complement, and angiogenesis and negatively associated with proliferative pathways and PD-1 signaling. These data indicate that the gene expression of the four identified macrophage clusters is phenotypically distinct with unique functionality.

We were unable to identify a distinct population of cells expressing *Lyve1*, possibly due to gene dropout (Supplementary Fig. S8A; ref. 49). Therefore, an alternative method was used to identify macrophages that exhibit a phenotype similar to that of *Lyve1-*expressing macrophages. In previous studies, we used bulk RNA sequencing of female mammary glands to identify 110 genes that were expressed significantly more highly in LYVE-1^+^ macrophages compared with LYVE-1^−^ macrophages (18). Together, these genes constitute the LYVE-1^+^ macrophage gene expression signature. In our scRNA-seq data, cluster 7 exhibited the highest levels of this LYVE-1^+^ macrophage gene expression signature suggesting cluster 7 represents the LYVE-1^+^ macrophage subset (Supplementary Fig. S8B). *Folr2*, which has been described as a marker of LYVE-1^+^ macrophages, is also preferentially found in cluster 7 and is reduced in *Lyve1^Cre^Csf1r^fl/fl^* mice, further confirming this finding (Fig. 5E; refs. 50, 51).

Tumors from *Lyve1^Cre^Csf1r^fl/fl^* mice exhibit a distinct reduction in cells associated with cluster 7, supporting our findings that this cluster likely represents the LYVE-1^+^ macrophage cluster (Fig. 5F). While we did find a reduction in the proportion of cells present in cluster 7, which is the cell population that putatively contains the LYVE-1–expressing macrophages, some cells remain in this cluster. This result may be due to incomplete genetic deletion or clustering of non-LYVE-1^+^ macrophages with similar phenotypic profiles.

Because we have defined a relationship between LYVE-1^+^ macrophages and ECM remodeling, GSEA was used to probe tissue remodeling pathways to compare macrophage clusters isolated from mammary tumors from *Lyve1^Cre^Csf1r^fl/fl^* and control *Csf1r^fl/fl^* mice. Upon LYVE-1^+^ macrophage depletion in the *Lyve1^Cre^Csf1r^fl/fl^* model, cluster 7 was found to be the only macrophage cluster negatively associated with glycosaminoglycan binding with statistical significance (Fig. 5G), consistent with a role for this cluster in ECM binding.

### Depletion of LYVE-1^+^ Macrophages Promotes a Shift Toward a Proinflammatory Environment

**FIGURE 6:**
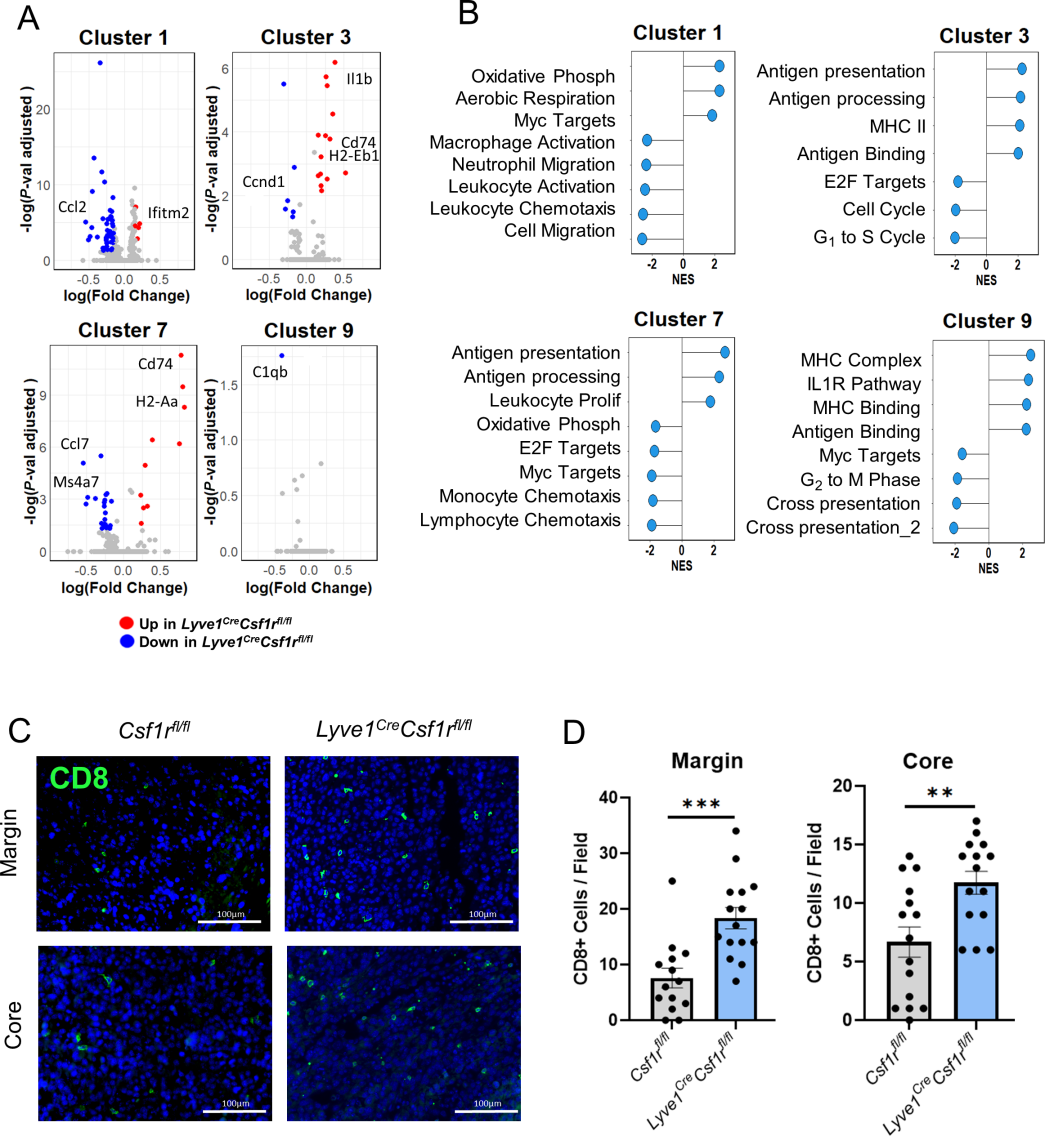
Depletion of LYVE-1^+^ macrophages promotes a shift toward a proinflammatory environment. **A,** Volcano plots of four macrophage clusters with log(fold change) of *Lyve1^Cre^Csf1r^fl/fl^* relative to *Csf1r^fl/fl^* and significance (*P <* 0.05) denoted as a red (upregulated) or blue (downregulated) coloration. **B,** Intracluster GSEA *Lyve1^Cre^Csf1r^fl/fl^* relative to *Csf1r^fl/fl^* macrophage clusters (NES adjusted *P*-value < 0.05) with detailed gene set names in Supplementary Table S2. Quantification (**C**) and representative images (**D**) of EO771 tumors from *Csf1r^fl/fl^* and *Lyve1^Cre^Csf1r^fl/fl^* mice immunostained for CD8a (green) and DAPI (blue). Each dot represents total CD8 count from one image, with five images taken per margin and five images taken per core of each tumor (*n* = 3). **, *P <* 0.01; ***, *P <* 0.001. Scale bars, 100 µm.

To assess the effects of LYVE-1^+^ macrophage depletion on the phenotypes of the remaining macrophages, differential gene expression analysis was performed comparing *Lyve1^Cre^Csf1r^fl/fl^* relative to *Csf1r^fl/fl^* samples (Supplementary Table S3). Analysis of genes that were significantly altered in each cluster suggested a phenotypic shift in two of the four macrophage clusters toward an immunostimulatory phenotype, as evidenced by the positive differential gene expression of *H2-Eb1* and *Cd74* (Fig. 6A). *H2-Eb1* positively contributes to antigen presentation, while *Cd74* contributes to both antigen presentation and cell survival (52, 53). Furthermore, upon LYVE-1^+^ macrophage depletion, a positive shift in *Il1b* expression is observed in cluster 3. Cluster 1 macrophages gain expression of *Ifitm2,* a monocyte recruiting cytokine, while the only gene differentially regulated in cluster 9 is the downregulation of *C1qb,* a gene involved in initiating complement (54, 55). These data suggest that loss of LYVE-1^+^ macrophages in the tumor microenvironment leads to a positive shift toward a proinflammatory phenotype in the remaining macrophages, indicating that when LYVE-1^+^ macrophages are present, they promote an anti-inflammatory environment in tumors.

Further analysis was performed using GSEA of the hallmark, M2, and M5 gene sets to compare macrophage clusters isolated from mammary tumors from *Lyve1^Cre^Csf1r^fl/fl^* and control *Csf1r^fl/fl^* mice to further characterize the distinct phenotypes of the four macrophage clusters (Fig. 6B; Supplementary Table S2). Upon LYVE-1^+^ macrophage depletion, cluster 1 is enriched in genes that are associated with oxidative phosphorylation, an anti-inflammatory macrophage process (56). In addition, cluster 1 is negatively enriched for a gene profile associated with leukocyte chemotaxis. Conversely, the cluster 3 gene expression profile is associated with antigen presentation upon the loss of LYVE-1^+^ macrophages and negatively associated with proliferation. Like cluster 3, the gene profiles of cluster 7 are also more associated with antigen presentation and less associated with proliferation and monocyte chemotaxis. Finally, cluster 9 gene profiles are aligned with antigen presentation genes, and negatively associated with proliferation. Thus, upon the depletion of LYVE-1^+^ macrophages, three of four macrophage subpopulations transcriptionally shift to a more antigen-presenting, less proliferative phenotype.

CD8^+^ T cells of cluster 8 were expanded in *Lyve1^Cre^Csf1r^fl/fl^* mice, indicating that the depletion of LYVE-1^+^ macrophages may impact the recruitment of adaptive immune cells (Fig. 5F). To validate the enrichment of CD8^+^ T cells of cluster 8 at the protein level, CD8^+^ cell count within the core and margin of tumors was analyzed by immunofluorescence. More CD8^+^ cells were found in tumors from *Lyve1^Cre^Csf1r^fl/fl^* mice at both the core and margin compared with control *Csf1r^fl/fl^* mice*,* corroborating conclusions from scRNA-seq (Fig. 6C and D). Thus, the depletion of LYVE-1^+^ macrophages results in increased CD8^+^ T-cell infiltration, which correlates with the reduction in primary tumor growth.

## Discussion

Using both *in vitro* and *in vivo* models, we demonstrate that LYVE-1^+^ macrophages are associated with ECM remodeling in both the normal mammary gland and in mammary tumors. Remodeling of the ECM is an essential homeostatic function in normal tissues and altered ECM remodeling is a key characteristic of tumor progression. Understanding the mechanisms by which tissue remodeling occurs is important for understanding both normal mammary gland homeostasis and tumor progression. Our findings suggest that LYVE-1^+^ macrophages are important regulators of HA within the stromal regions of the female mammary gland and mammary tumors. LYVE-1^+^ macrophages have also been identified in numerous tissues including the lung and aorta, as well as murine models of cancers such as MMTV-PyMT and melanoma tumors (11–13, 17). In a murine model of mammary adenocarcinoma, LYVE-1^+^ macrophages were found to localize near blood vessels and help maintain perivascular pericyte-like cells throughout tumor development (21). In addition, PDPN-expressing macrophages, a subset of which express LYVE-1, localize near lymphatic regions and contribute to lymphangiogenesis and lymphoinvasion (14, 57). We demonstrate here an additional subset of LYVE-1^+^ macrophages that can be found in association with HA-rich regions associated with the mammary tumor margin. This localization pattern is consistent with observations in other models such as B16F1 melanoma, where LYVE-1^+^ macrophages have been found in the periphery (17). Timperi and colleagues identify an immunosuppressive LYVE-1^+^ macrophage subset in human patients with breast cancer, localized to the tumor stroma (38). This macrophage subset is specifically enriched in patients resistant to immune checkpoint blockade; however, patient outcome was not evaluated in this study. In addition, FOLR2^+^ macrophages, which also express LYVE-1, have been found in human mammary tissue and tumors (50). FOLR2^+^ macrophages were identified in perivascular regions of the tumor stroma and their infiltration correlates with better patient survival. These findings raise the interesting possibility that there may be functional heterogeneity within the LYVE-1^+^ macrophage population based on differences in their specific histologic localization within normal tissue and tumors.

To examine the function of LYVE-1^+^ macrophages we utilized the *Lyve1^Cre^Csf1r^fl/fl^* genetic model of LYVE-1^+^ macrophage depletion. We found a significant depletion of LYVE-1^+^ macrophages, with no significant impact on the presence of LYVE-1^+^CD45^−^ LECs. However, it is important to note that our studies did not assess any impact on structure or function of LECs, which could be addressed in future studies. Furthermore, whether the loss of LYVE-1^+^ macrophages leads to a compensatory increase in other macrophage subsets in this model has not been investigated. We observed a small reduction in total macrophage count by flow cytometry (Supplementary Fig. S1B) and cell count by scRNA-seq (Supplementary Table S4), suggesting incomplete compensation. Regardless of these potential caveats with the model, we felt that the significant depletion of LYVE-1^+^ macrophages was sufficient for further functional studies focused on their role in mammary tumor growth.

To determine the functional contributions of LYVE-1^+^ macrophages to tumor growth and progression, the ability of tumors to grow in the *Lyve1^Cre^Csf1r^fl/fl^* model was assessed. Using two distinct orthotopic mammary tumor models in immune competent mice, we observed a decrease in tumor growth in the absence of LYVE-1^+^ macrophages, and using the EO771 model, a reduction in lung colonization. This reduction correlated with an increase in tumor-associated HA. The presence and function of HA within the tumor microenvironment is complex. HA accumulation has been associated with tumor suppression in both cell line and *in vivo* models. A notable example of this is the naked mole rat model, in which accumulation of HMW-HA is linked with suppression of tumor growth (58–61). In contrast, high levels of HA are associated with poor prognosis in patients with breast cancer (62, 63). Furthermore, studies have defined both protumorigenic and antitumorigenic roles for HA depending upon the molecular weight of HA, as well as tumor type and stage (60, 62). Thus, the functions of HA in the tumor microenvironment are highly context dependent. The accumulation of HA observed in the context of LYVE-1^+^ macrophage depletion is consistent with a potential tumor suppressive role, possibly by acting as a barrier to tumor cell migration or enhancing sequestration of growth factors.

Depletion of LYVE-1^+^ macrophages correlates with increased apoptosis compared with control mice specifically at the margin, with no changes observed between genotypes in the tumor core. Interestingly, scRNA-seq analysis demonstrates that *C1q* genes are specifically enriched in the LYVE-1^+^ macrophage cluster. This raises the intriguing possibility that LYVE-1^+^ macrophages may be involved in apoptotic clearance. *C1qa*, *C1qb*, and *C1qc* genes make up the globular domain of the C1q protein, an initiating factor in the classical complement cascade (64). The complement system has many functions in innate immunity including the clearance of apoptotic debris, thus it is possible that the increase in apoptotic cells in the tumor margin may be due to reduced apoptotic cell clearance in the absence of LYVE-1^+^ macrophages (65). In addition, increased apoptosis at the tumor margin in the depletion model could suggest that LYVE-1^+^ macrophages produce factors that promote survival specifically at the tumor margin. Notably, several genes that encode growth factors that are known to be associated with cell survival, including *Tgfb1*, *Pdgfb*, and *Pgf* are enriched in this cluster (Supplementary Table S1; refs. 66–68).

LYVE-1 is a receptor for HA and is known to promote HA internalization by LECs (12). In our previous studies, we demonstrated that global macrophage depletion led to increased HA accumulation in the mammary gland (18). On the basis of these findings, we concluded that macrophages contribute to HA turnover in the mammary gland stroma. However, because we used a CSF1R inhibitor to deplete macrophages, this strategy did not specifically describe a role for LYVE-1^+^ macrophages in this process. We demonstrate here that selective depletion of LYVE-1^+^ macrophages leads to increased HA levels in the mammary glands, demonstrating that LYVE-1^+^ macrophages are indeed responsible for contributing to HA turnover in the mammary gland. Furthermore, we determine that blocking LYVE-1 reduces HA binding in macrophages. Consistent with this finding, Voisin and colleagues recently have shown that hypodermal macrophages, a macrophage subset that expresses both LYVE-1 and FOLR2, internalize HA in a LYVE-1–dependent manner (33). Our previous studies also demonstrated that global macrophage depletion resulted in collagen accumulation in the mammary stroma (18). However, a similar increase in collagen levels was not observed upon depletion of the LYVE-1^+^ macrophage population alone. Furthermore, Voisin and colleagues find a decrease in collagen in the skin upon the deletion of hypodermal macrophages. Interestingly, in our depletion model, fibronectin protein levels were unchanged. Thus, while LYVE-1 macrophages have a clear role in modulating HA across a number of experimental models, further studies are required to determine whether LYVE-1^+^ macrophages modulate other ECM-associated factors such as collagen or fibronectin.

The ECM remodeling phenotype of LYVE-1^+^ macrophages that we previously identified in the normal mammary gland (18) was found to be conserved in tumor-associated macrophages as identified by scRNA-seq analysis. Furthermore, *in vitro* studies suggest that LYVE-1 expression correlates with the ability of macrophages to internalize and degrade HA, and blocking LYVE-1 reduces the ability of macrophages to bind HA. In conjunction with the increased HA accumulation in the tumors from LYVE-1^+^ macrophage-depleted mice, these findings suggest that LYVE-1^+^ macrophages contribute to HA degradation in the tumor microenvironment. Further studies are needed to assess expression levels of hyaluronidases and HA synthases in primary tumor-derived LYVE-1^+^ macrophages. Notably, previous work from our lab has found *Hyal* genes upregulated in macrophages compared with other cell types, specifically tumor cells and fibroblasts, in mammary tumor models suggesting a potential role for macrophages in directly modulating HA levels (27). However, it is also possible that LYVE-1^+^ macrophages act in concert with other cell types, such as fibroblasts, to drive HA turnover. HA degradation by hyaluronidases and ROS can lead to the generation of LMW-HA fragments, which have been shown to promote tumor cell proliferation (69). Thus, while not specifically addressed by this study, it is possible that LYVE-1^+^ macrophages contribute to the generation of tumor supportive LMW-HA fragments in the tumor microenvironment and that depletion of these macrophages leads to a reduction in tumor-promoting LMW fragments and accumulation of tumor suppressive HMW-HA. Therefore, further studies are needed to evaluate the contributions of LYVE-1^+^ macrophages to HA fragment size within tumors.

In addition to tissue remodeling, the results from the scRNA-seq analysis demonstrate that LYVE-1^+^ macrophages promote an anti-inflammatory tumor microenvironment. When LYVE-1^+^ macrophages were depleted, a distinct phenotypic shift occurred, shifting the transcriptional profiles of three of the four macrophage clusters to a proinflammatory phenotype. The LYVE-1^+^ macrophage cluster is enriched for immunosuppressive markers such as *Apoe* and *Ctsb* (70, 71). Furthermore, the LYVE-1^+^ macrophage cluster is heavily enriched for *C1q* genes, which are associated with an immunosuppressed tumor microenvironment in human patients with colorectal cancer (72). Finally, more CD8^+^ T cells were found in both the margin and core of tumors with LYVE-1^+^ macrophages depleted. In human patients with breast cancer, CD8^+^ T-cell infiltration correlates with improved patient survival (73). Together, these findings suggest that in addition to ECM modulation, LYVE-1^+^ macrophages also serve an anti-inflammatory function within the tumor microenvironment.

In summary, this study describes the localization, phenotype, and function of LYVE-1^+^ macrophages in the female mouse mammary gland and in mouse mammary tumors. Using a combination of *in vitro* and *in vivo* models, our findings show that LYVE-1^+^ macrophages regulate HA turnover and LYVE-1 expression correlates with HA binding, internalization, and degradation. Furthermore, the results demonstrate that the LYVE-1^+^ macrophage population maintains an anti-inflammatory tumor microenvironment, supports tumor growth, and restricts CD8^+^ T-cell count. These studies reveal a unique phenotype for LYVE-1^+^ macrophages in both the normal tissue and tumor setting. Despite their importance in tumor progression, targeting macrophages in the clinic has been challenging (74). Lack of efficacy of targeting macrophages in patients with cancer may be due to many factors, including the functional heterogeneity of macrophages and lack of specificity of macrophage targeting drugs. Understanding phenotypic macrophage subsets, such as the tumor-supporting LYVE-1^+^ macrophages, may provide additional viable targets for improved cancer therapeutics.

## Supplementary Material

Supplementary Table 1 Table depicting differential gene expression data comparing each cluster relative to all other clusters within tumors derived from Csfrfl/fl mice.

Supplementary Table 2 Table depicting shortened GSEA terms and corresponding full GSEA name referenced in Figure 5D and 6B.

Supplementary Table 3 Table depicting differential gene expression data comparing each Lyve1CreCsfrfl/fl cluster relative to its corresponding cluster in Csfrfl/fl mice.

Supplementary Table 4 Table depicting total cell counts for each scRNA-seq cluster in both Lyve1CreCsfrfl/fl and Csfrfl/fl mice.

Supplementary Figure 1 Figure S1 depicts representative flow cytometry gating and macrophage counts

Supplementary Figure 2 Figure S2 depicts representative mammary gland flow cytometry gating

Supplementary Figure 3 Figure S3 depicts an ELISA analyzing collagen content in mammary glands

Supplementary Figure 4 Figure S4 depicts representative flow cytometry gating for EO771 tumors

Supplementary Figure 5 Figure S5 depicts tumor growth rates

Supplementary Figure 6 Figure S6 depicts scRNA-seq immune cell feature plots.

Supplementary Figure 7 Figure S7 depicts an scRNA-seq heatmap of the top 10 genes per cluster

Supplementary Figure 8 Figure S8 depicts the LYVE-1+ macrophage gene signature feature plots
