# Supplementary material for: LYVE-1–expressing Macrophages Modulate the Hyaluronan-containing Extracellular Matrix in the Mammary Stroma and Contribute to Mammary Tumor Growth
Source: Cancer Res Commun. 2024 May 31;4(5):1380–97. doi: 10.1158/2767-9764.CRC-24-0205 (PMC11141485; doi:10.1158/2767-9764.CRC-24-0205)
Supplement: Supplementary Figure 1 — Figure S1 depicts representative flow cytometry gating and macrophage counts [file crc-24-0205-s05.pdf]

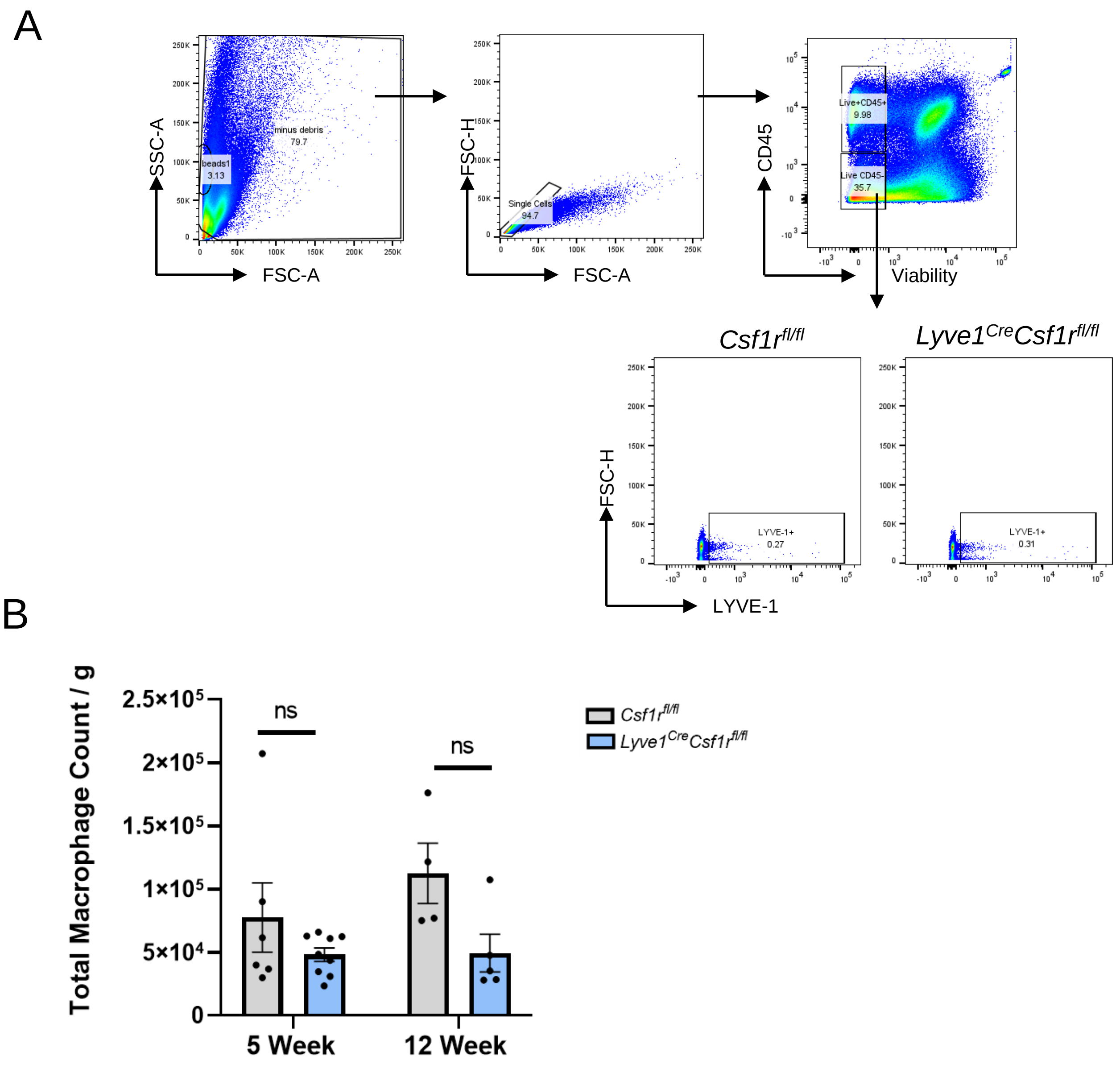

Figure S1

Representative flow cytometry gating and macrophage counts

(A) Representative flow cytometry gating for CD45-LYVE-1^{+} LECs. (B) Total CD45^{+}F4/80^{+}CD11b^{+} macrophage count normalized to mammary gland weight from 5-week and 12-week female *Csf1r*$^{fl/fl}$ (grey) and *Lyve1*^{Cre}*Csf1r*$^{fl/fl}$ (blue) mice. Each dot represents one mouse.
