# Supplementary material for: LYVE-1–expressing Macrophages Modulate the Hyaluronan-containing Extracellular Matrix in the Mammary Stroma and Contribute to Mammary Tumor Growth
Source: Cancer Res Commun. 2024 May 31;4(5):1380–97. doi: 10.1158/2767-9764.CRC-24-0205 (PMC11141485; doi:10.1158/2767-9764.CRC-24-0205)
Supplement: Supplementary Figure 2 — Figure S2 depicts representative mammary gland flow cytometry gating [file crc-24-0205-s06.pdf]

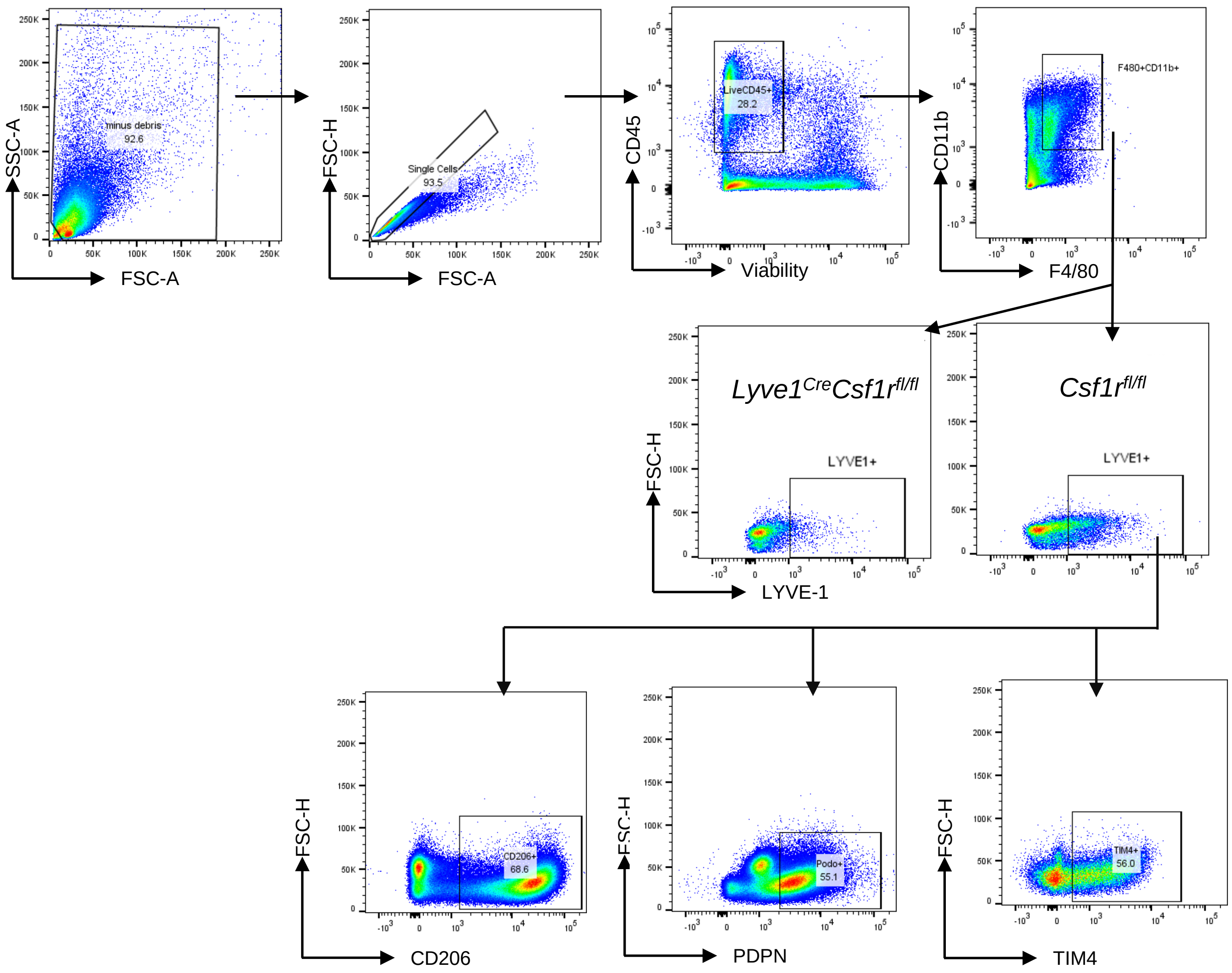

Figure S2

Representative mammary gland flow cytometry gating

Representative flow cytometry gating for CD45^{+}F4/80^{+}CD11b^{+}LYVE-1^{+} macrophages coexpressing CD206, PDPN, and TIM4 in mammary glands from 5-week *Csf1r*$^{fl/fl}$ and *Lyve1*^{Cre}*Csf1r*$^{fl/fl}$ mice.
