# Supplementary material for: LYVE-1–expressing Macrophages Modulate the Hyaluronan-containing Extracellular Matrix in the Mammary Stroma and Contribute to Mammary Tumor Growth
Source: Cancer Res Commun. 2024 May 31;4(5):1380–97. doi: 10.1158/2767-9764.CRC-24-0205 (PMC11141485; doi:10.1158/2767-9764.CRC-24-0205)
Supplement: Supplementary Figure 3 — Figure S3 depicts an ELISA analyzing collagen content in mammary glands [file crc-24-0205-s07.pdf]

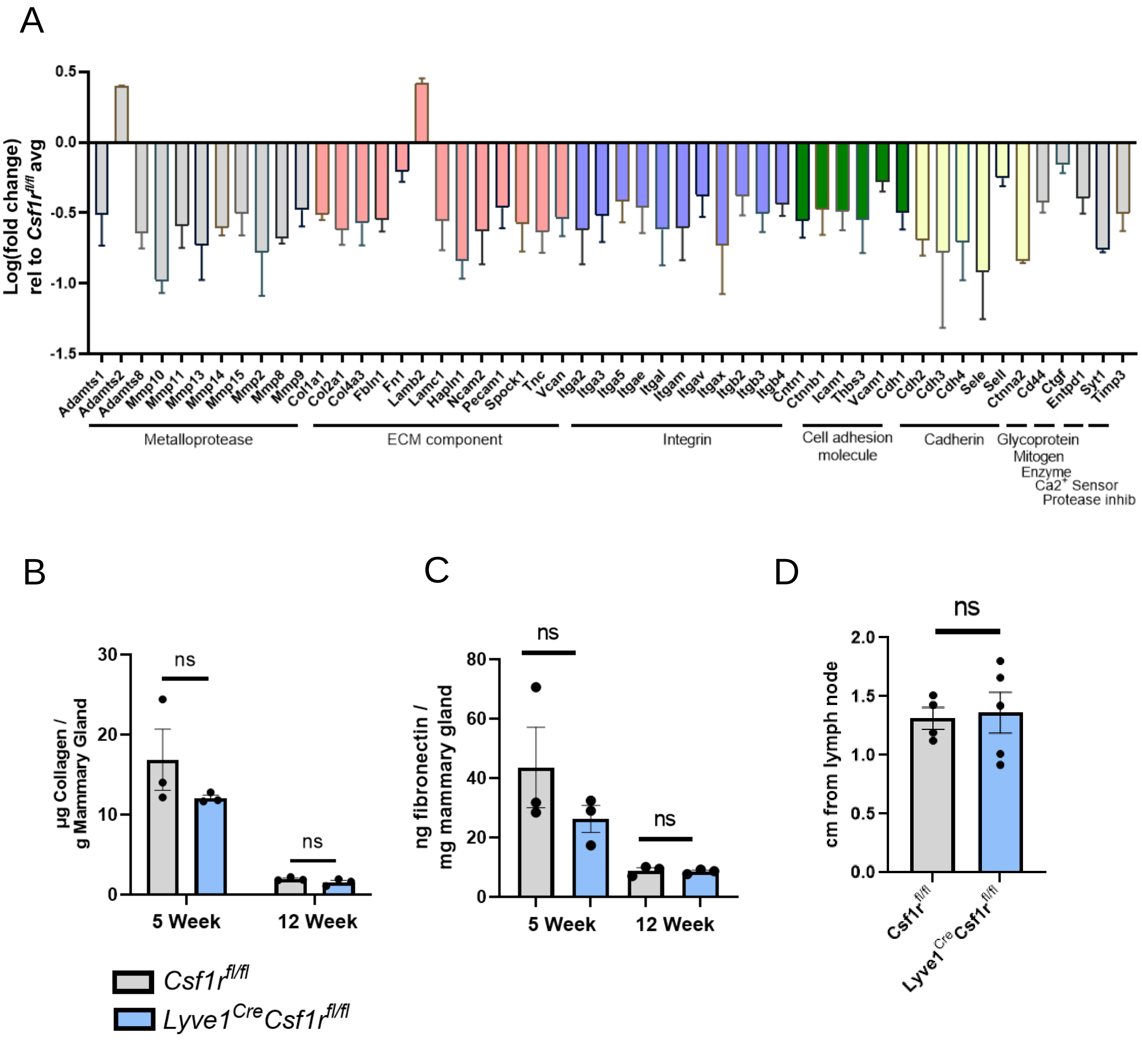

Figure S3

ELISA analyzing collagen content in mammary glands

(A) Female mammary glands assessed by ECM qRT-PCR array of significantly differentially expressed genes (n=3 per genotype). (B) Mouse Pro-Collagen I alpha 1 protein content of female mammary glands normalized to weight. (C) Mouse fibronectin protein content of female mammary glands normalized to weight. (D) Distance from lymph node to terminal end bud in mammary gland whole mounts from 7-week female *Csf1r*$^{fl/fl}$ and *Lyve1*^{Cre}*Csf1r*$^{fl/fl}$ mice. Each dot represents one mouse.
