# Supplementary material for: LYVE-1–expressing Macrophages Modulate the Hyaluronan-containing Extracellular Matrix in the Mammary Stroma and Contribute to Mammary Tumor Growth
Source: Cancer Res Commun. 2024 May 31;4(5):1380–97. doi: 10.1158/2767-9764.CRC-24-0205 (PMC11141485; doi:10.1158/2767-9764.CRC-24-0205)
Supplement: Supplementary Figure 4 — Figure S4 depicts representative flow cytometry gating for EO771 tumors [file crc-24-0205-s08.pdf]

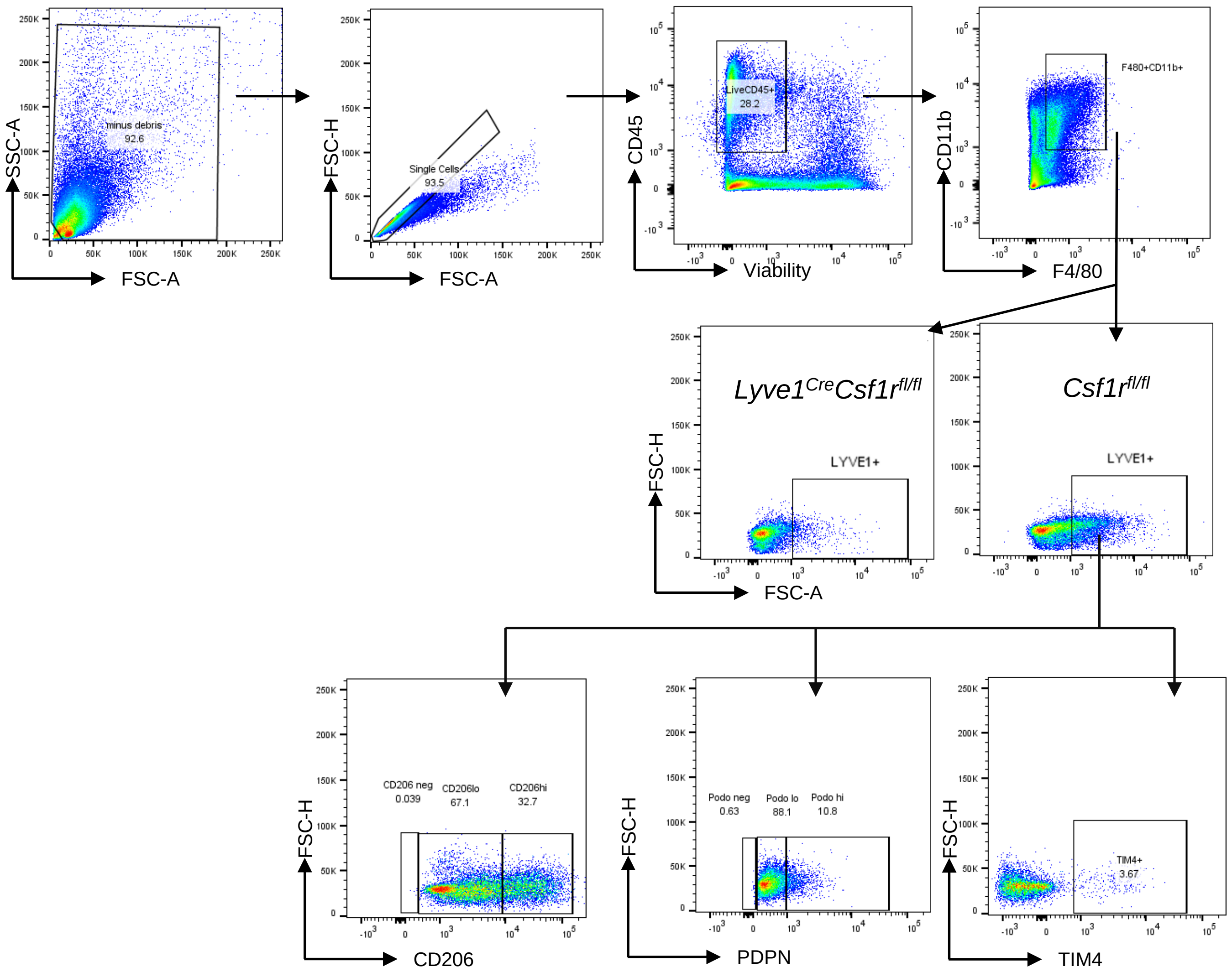

Figure S4

Representative flow cytometry gating for EO771 tumors.
Representative flow cytometry gating for CD45$^+$F4/80$^+$CD11b$^+$LYVE-1$^+$ macrophages coexpressing CD206, PDPN, and TIM4 in EO771 tumors from *Csf1r*$^{fl/fl}$ and *Lyve1*^{Cre}*Csf1r*$^{fl/fl}$ mice.
