# Supplementary material for: LYVE-1–expressing Macrophages Modulate the Hyaluronan-containing Extracellular Matrix in the Mammary Stroma and Contribute to Mammary Tumor Growth
Source: Cancer Res Commun. 2024 May 31;4(5):1380–97. doi: 10.1158/2767-9764.CRC-24-0205 (PMC11141485; doi:10.1158/2767-9764.CRC-24-0205)
Supplement: Supplementary Figure 5 — Figure S5 depicts tumor growth rates [file crc-24-0205-s09.pdf]

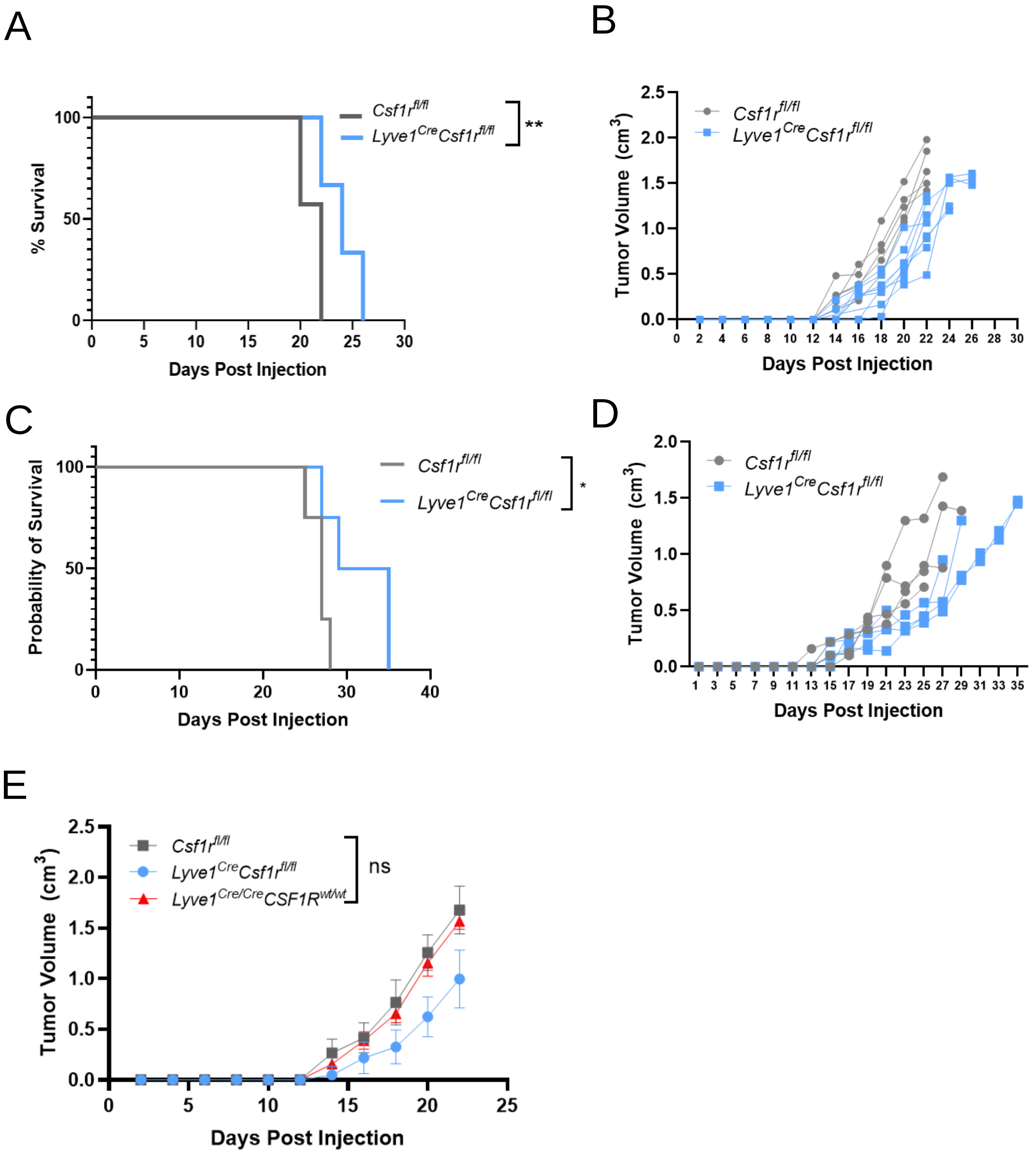


**Figure S5**

**Tumor growth curve**

(A) Kaplan-Meier plot and (B) growth curve through end point for EO771 tumors in *Csf1r*$^{fl/fl}$ (grey, n=4) *Lyve1*$^{Cre}$*Csf1r*$^{fl/fl}$ (blue, n=4). (C) Kaplan-Meier plot and (D) growth curve through end point for WNT-4226 tumors in *Csf1r*$^{fl/fl}$ (grey, n=4) *Lyve1*$^{Cre}$*Csf1r*$^{fl/fl}$ (blue, n=4). (E) EO771 tumor growth in *Csf1r*$^{fl/fl}$ (grey, n=4) *Lyve1*$^{Cre}$*Csf1r*$^{fl/fl}$ (blue, n=4) and *Lyve1*$^{Cre/Cre}$*Csf1r*$^{wt/wt}$ *(*red, n=3) mice.
