# Supplementary material for: LYVE-1–expressing Macrophages Modulate the Hyaluronan-containing Extracellular Matrix in the Mammary Stroma and Contribute to Mammary Tumor Growth
Source: Cancer Res Commun. 2024 May 31;4(5):1380–97. doi: 10.1158/2767-9764.CRC-24-0205 (PMC11141485; doi:10.1158/2767-9764.CRC-24-0205)
Supplement: Supplementary Figure 6 — Figure S6 depicts scRNA-seq immune cell feature plots. [file crc-24-0205-s10.pdf]

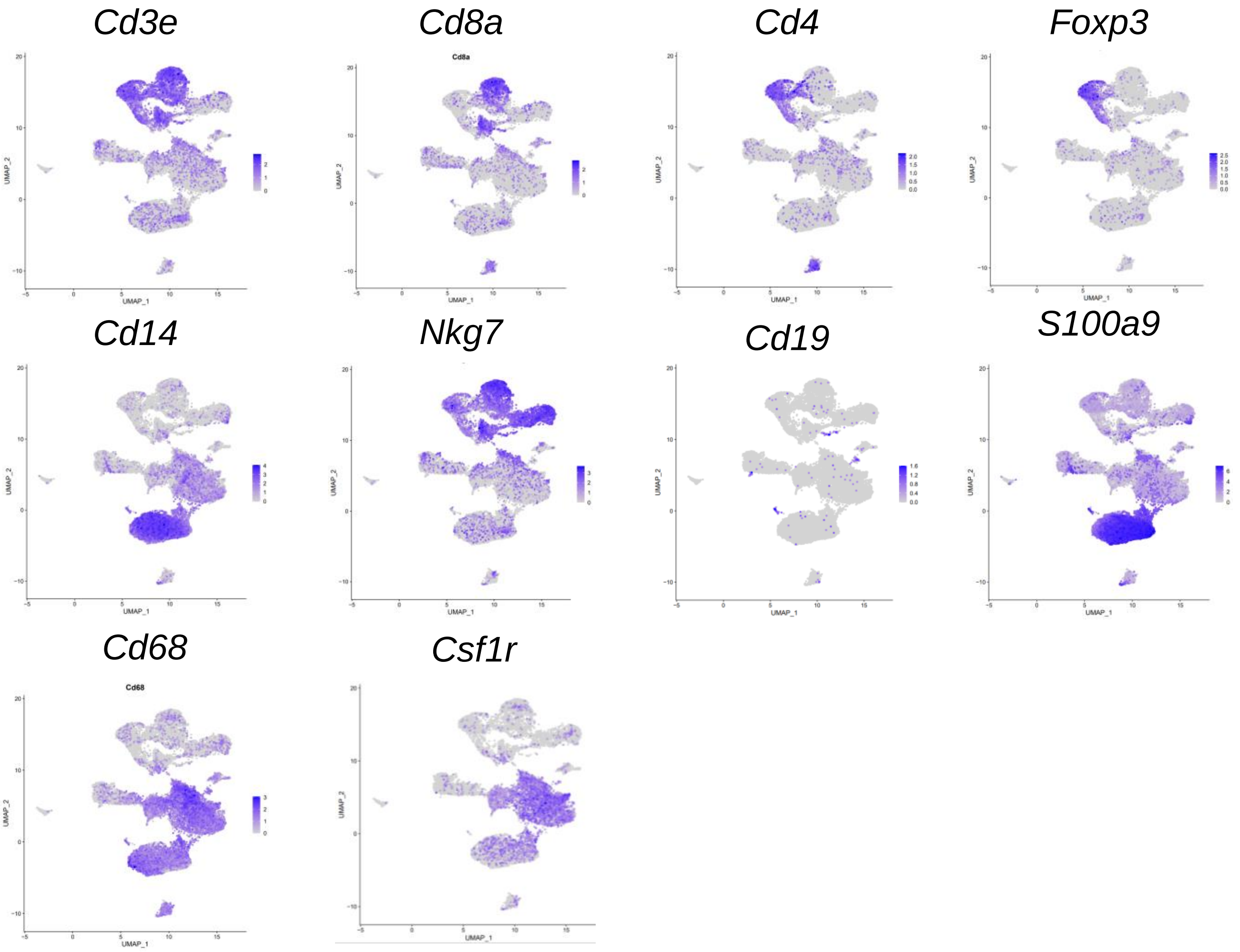

Figure S6

scRNA-seq Immune Cell Feature Plots.

Feature plots of immune cell markers *Cd3e*, *Cd8a*, *Cd4*, *Foxp3*, *Cd14*, *Nkg7*, *Cd19*, and *S100a9* from EO771 tumors from *Csf1r*$^{fl/fl}$ mice.
