# Supplementary material for: LYVE-1–expressing Macrophages Modulate the Hyaluronan-containing Extracellular Matrix in the Mammary Stroma and Contribute to Mammary Tumor Growth
Source: Cancer Res Commun. 2024 May 31;4(5):1380–97. doi: 10.1158/2767-9764.CRC-24-0205 (PMC11141485; doi:10.1158/2767-9764.CRC-24-0205)
Supplement: Supplementary Figure 7 — Figure S7 depicts an scRNA-seq heatmap of the top 10 genes per cluster [file crc-24-0205-s11.pdf]

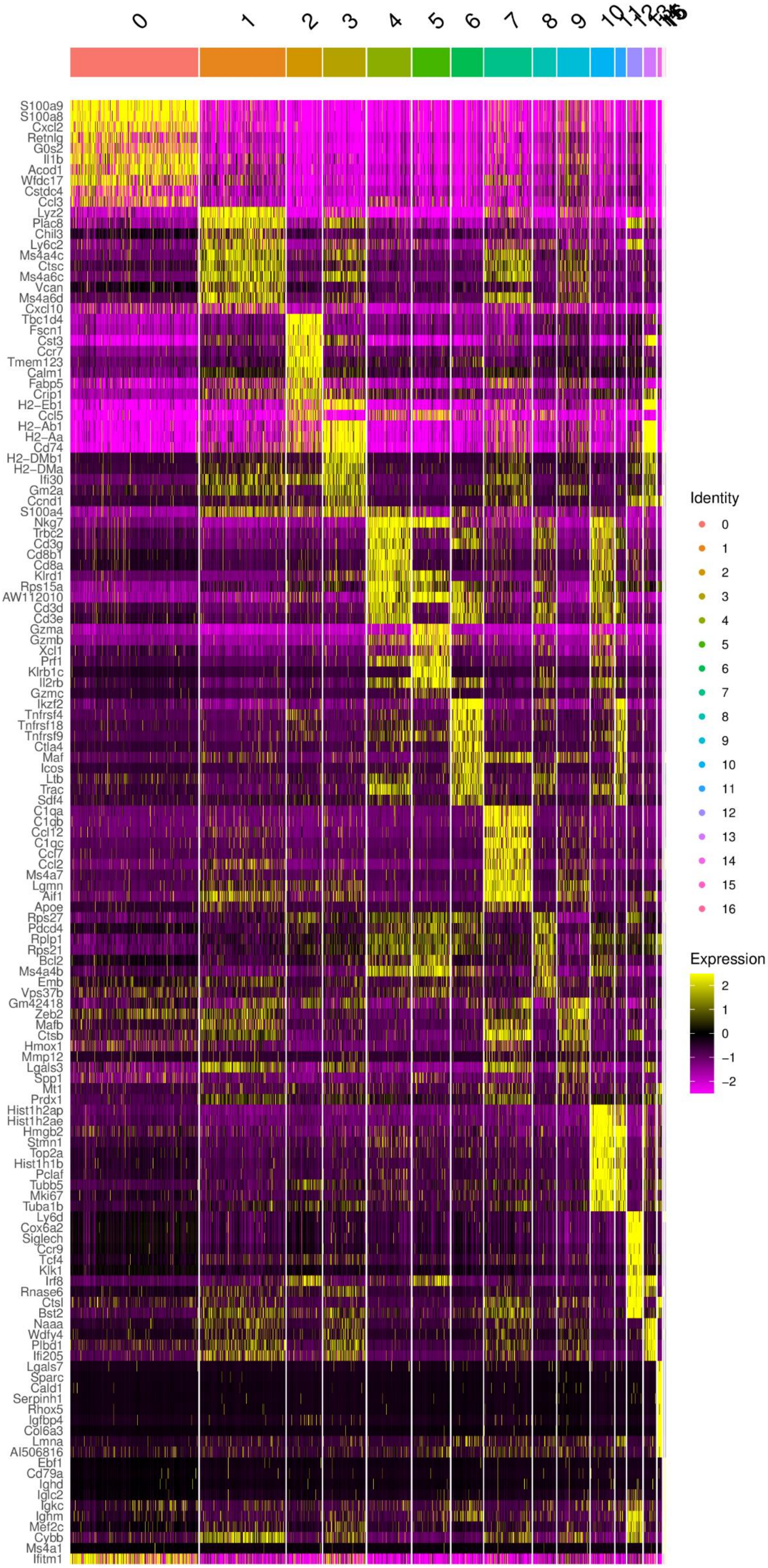

Figure S7

scRNA-seq heatmap of top 10 genes per cluster.

Heatmap of top 10 genes per cluster from EO771 tumors grown in *Csf1r*$^{fl/fl}$ mice.
