# Supplementary material for: LYVE-1–expressing Macrophages Modulate the Hyaluronan-containing Extracellular Matrix in the Mammary Stroma and Contribute to Mammary Tumor Growth
Source: Cancer Res Commun. 2024 May 31;4(5):1380–97. doi: 10.1158/2767-9764.CRC-24-0205 (PMC11141485; doi:10.1158/2767-9764.CRC-24-0205)
Supplement: Supplementary Figure 8 — Figure S8 depicts the LYVE-1+ macrophage gene signature feature plots [file crc-24-0205-s12.pdf]

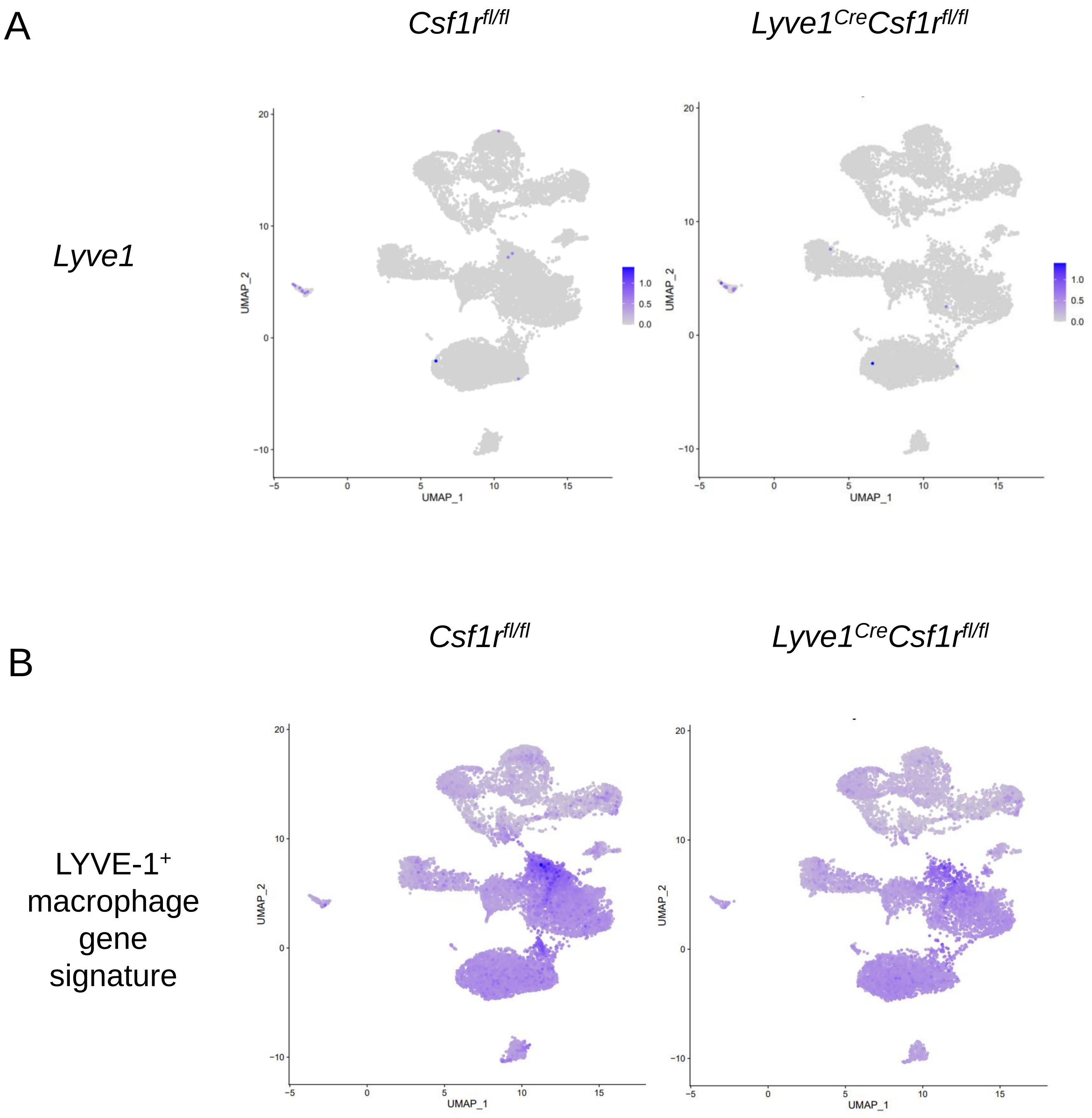

Figure S8

LYVE-1$^+$ macrophage gene signature feature plots.

(A) Feature plot of *Lyve1* in *Csf1r$^{fl/fl}$* and *Lyve1^{Cre}Csf1r$^{fl/fl}$* samples. (B) Feature plot of LYVE-1$^+$ macrophage gene signature from *Csf1r$^{fl/fl}$* and *Lyve1^{Cre}Csf1r$^{fl/fl}$* samples.
